# Supplementary material for: Incentives and barriers to private finance for forest and landscape restoration
Source: Nat Ecol Evol. 2023 May 8;7(5):707–15. doi: 10.1038/s41559-023-02037-5 (PMC10172125; doi:10.1038/s41559-023-02037-5)
Supplement: Supplementary file 1 — Supplementary Appendices A–C. [file 41559_2023_2037_MOESM1_ESM.pptx]

## Slide 1
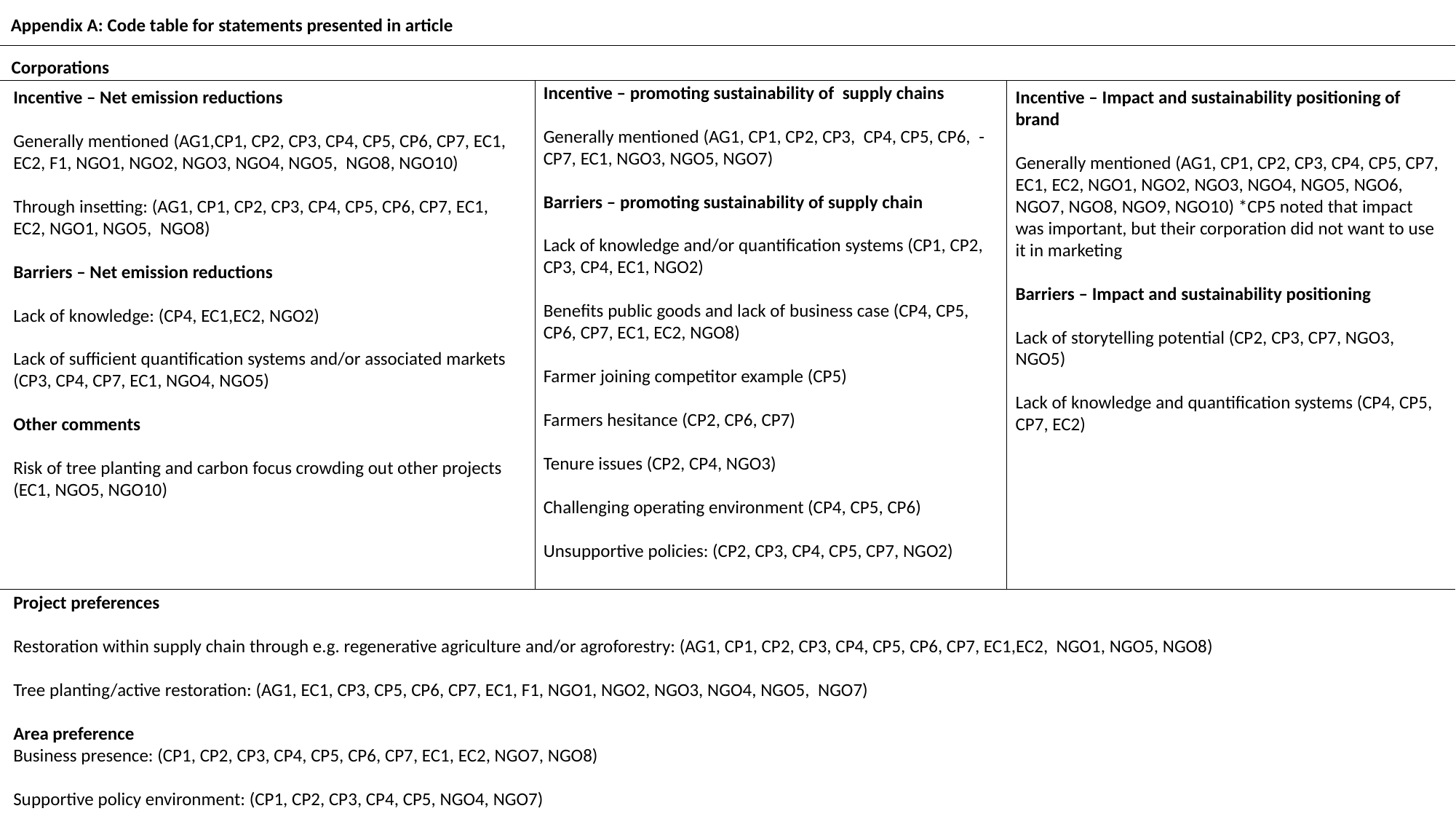

Appendix A: Code table for statements presented in article
Corporations
Incentive – promoting sustainability of supply chains
Generally mentioned (AG1, CP1, CP2, CP3, CP4, CP5, CP6, -CP7, EC1, NGO3, NGO5, NGO7)
Barriers – promoting sustainability of supply chain
Lack of knowledge and/or quantification systems (CP1, CP2, CP3, CP4, EC1, NGO2)
Benefits public goods and lack of business case (CP4, CP5, CP6, CP7, EC1, EC2, NGO8)
Farmer joining competitor example (CP5)
Farmers hesitance (CP2, CP6, CP7)
Tenure issues (CP2, CP4, NGO3)
Challenging operating environment (CP4, CP5, CP6)
Unsupportive policies: (CP2, CP3, CP4, CP5, CP7, NGO2)
Incentive – Impact and sustainability positioning of brand
Generally mentioned (AG1, CP1, CP2, CP3, CP4, CP5, CP7, EC1, EC2, NGO1, NGO2, NGO3, NGO4, NGO5, NGO6, NGO7, NGO8, NGO9, NGO10) *CP5 noted that impact was important, but their corporation did not want to use it in marketing
Barriers – Impact and sustainability positioning
Lack of storytelling potential (CP2, CP3, CP7, NGO3, NGO5)
Lack of knowledge and quantification systems (CP4, CP5, CP7, EC2)
Incentive – Net emission reductions
Generally mentioned (AG1,CP1, CP2, CP3, CP4, CP5, CP6, CP7, EC1, EC2, F1, NGO1, NGO2, NGO3, NGO4, NGO5, NGO8, NGO10)
Through insetting: (AG1, CP1, CP2, CP3, CP4, CP5, CP6, CP7, EC1, EC2, NGO1, NGO5, NGO8)
Barriers – Net emission reductions
Lack of knowledge: (CP4, EC1,EC2, NGO2)
Lack of sufficient quantification systems and/or associated markets (CP3, CP4, CP7, EC1, NGO4, NGO5)
Other comments
Risk of tree planting and carbon focus crowding out other projects (EC1, NGO5, NGO10)
Project preferences
Restoration within supply chain through e.g. regenerative agriculture and/or agroforestry: (AG1, CP1, CP2, CP3, CP4, CP5, CP6, CP7, EC1,EC2, NGO1, NGO5, NGO8)
Tree planting/active restoration: (AG1, EC1, CP3, CP5, CP6, CP7, EC1, F1, NGO1, NGO2, NGO3, NGO4, NGO5, NGO7)
Area preference
Business presence: (CP1, CP2, CP3, CP4, CP5, CP6, CP7, EC1, EC2, NGO7, NGO8)
Supportive policy environment: (CP1, CP2, CP3, CP4, CP5, NGO4, NGO7)

## Slide 2
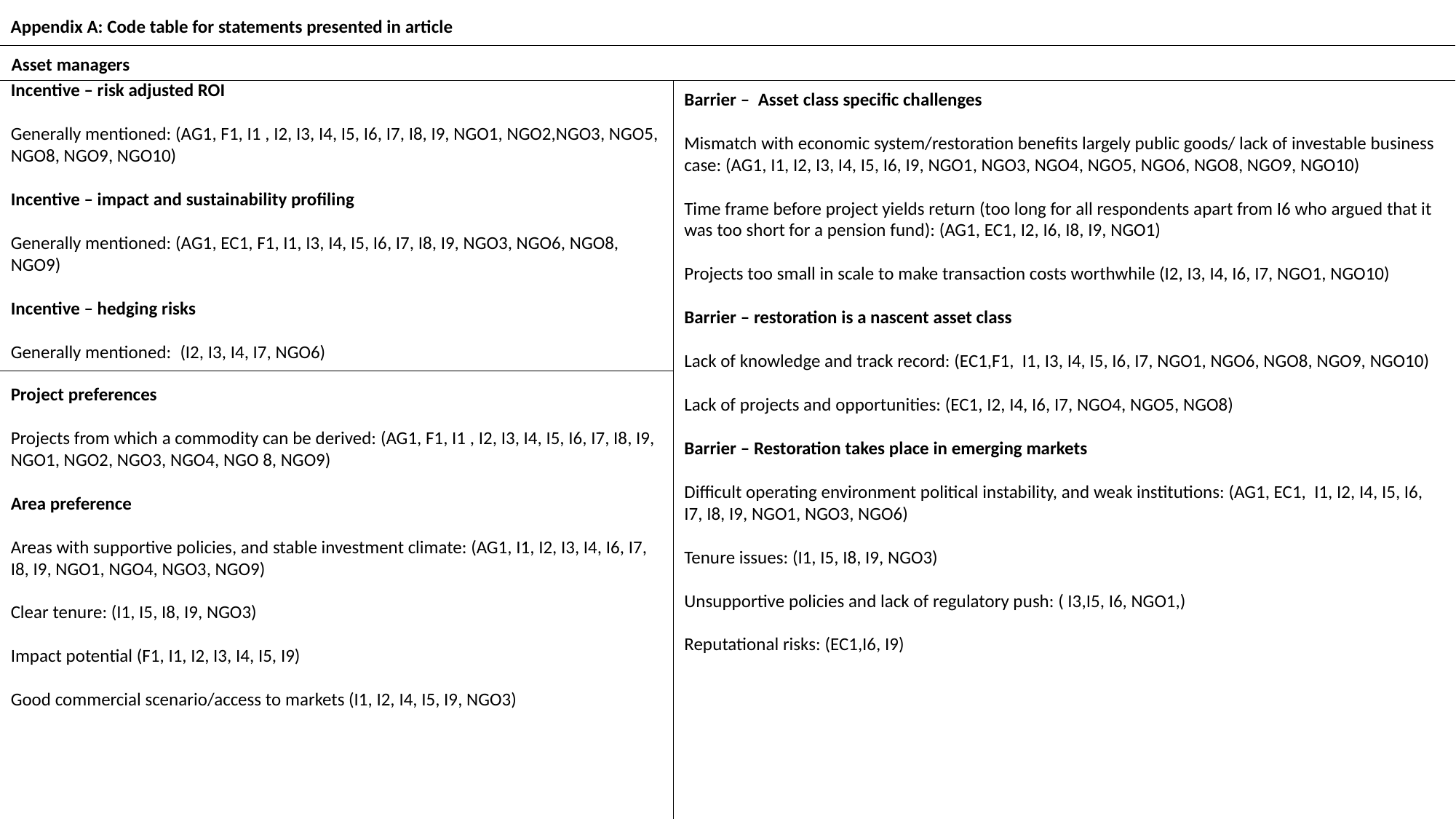

Appendix A: Code table for statements presented in article
Asset managers
Incentive – risk adjusted ROI
Generally mentioned: (AG1, F1, I1 , I2, I3, I4, I5, I6, I7, I8, I9, NGO1, NGO2,NGO3, NGO5, NGO8, NGO9, NGO10)
Incentive – impact and sustainability profiling
Generally mentioned: (AG1, EC1, F1, I1, I3, I4, I5, I6, I7, I8, I9, NGO3, NGO6, NGO8, NGO9)
Incentive – hedging risks
Generally mentioned:  (I2, I3, I4, I7, NGO6)
Barrier – Asset class specific challenges
Mismatch with economic system/restoration benefits largely public goods/ lack of investable business case: (AG1, I1, I2, I3, I4, I5, I6, I9, NGO1, NGO3, NGO4, NGO5, NGO6, NGO8, NGO9, NGO10)
Time frame before project yields return (too long for all respondents apart from I6 who argued that it was too short for a pension fund): (AG1, EC1, I2, I6, I8, I9, NGO1)
Projects too small in scale to make transaction costs worthwhile (I2, I3, I4, I6, I7, NGO1, NGO10)
Barrier – restoration is a nascent asset class
Lack of knowledge and track record: (EC1,F1, I1, I3, I4, I5, I6, I7, NGO1, NGO6, NGO8, NGO9, NGO10)
Lack of projects and opportunities: (EC1, I2, I4, I6, I7, NGO4, NGO5, NGO8)
Barrier – Restoration takes place in emerging markets
Difficult operating environment political instability, and weak institutions: (AG1, EC1, I1, I2, I4, I5, I6, I7, I8, I9, NGO1, NGO3, NGO6)
Tenure issues: (I1, I5, I8, I9, NGO3)
Unsupportive policies and lack of regulatory push: ( I3,I5, I6, NGO1,)
Reputational risks: (EC1,I6, I9)
Project preferences
Projects from which a commodity can be derived: (AG1, F1, I1 , I2, I3, I4, I5, I6, I7, I8, I9, NGO1, NGO2, NGO3, NGO4, NGO 8, NGO9)
Area preference
Areas with supportive policies, and stable investment climate: (AG1, I1, I2, I3, I4, I6, I7, I8, I9, NGO1, NGO4, NGO3, NGO9)
Clear tenure: (I1, I5, I8, I9, NGO3)
Impact potential (F1, I1, I2, I3, I4, I5, I9)
Good commercial scenario/access to markets (I1, I2, I4, I5, I9, NGO3)

## Slide 3
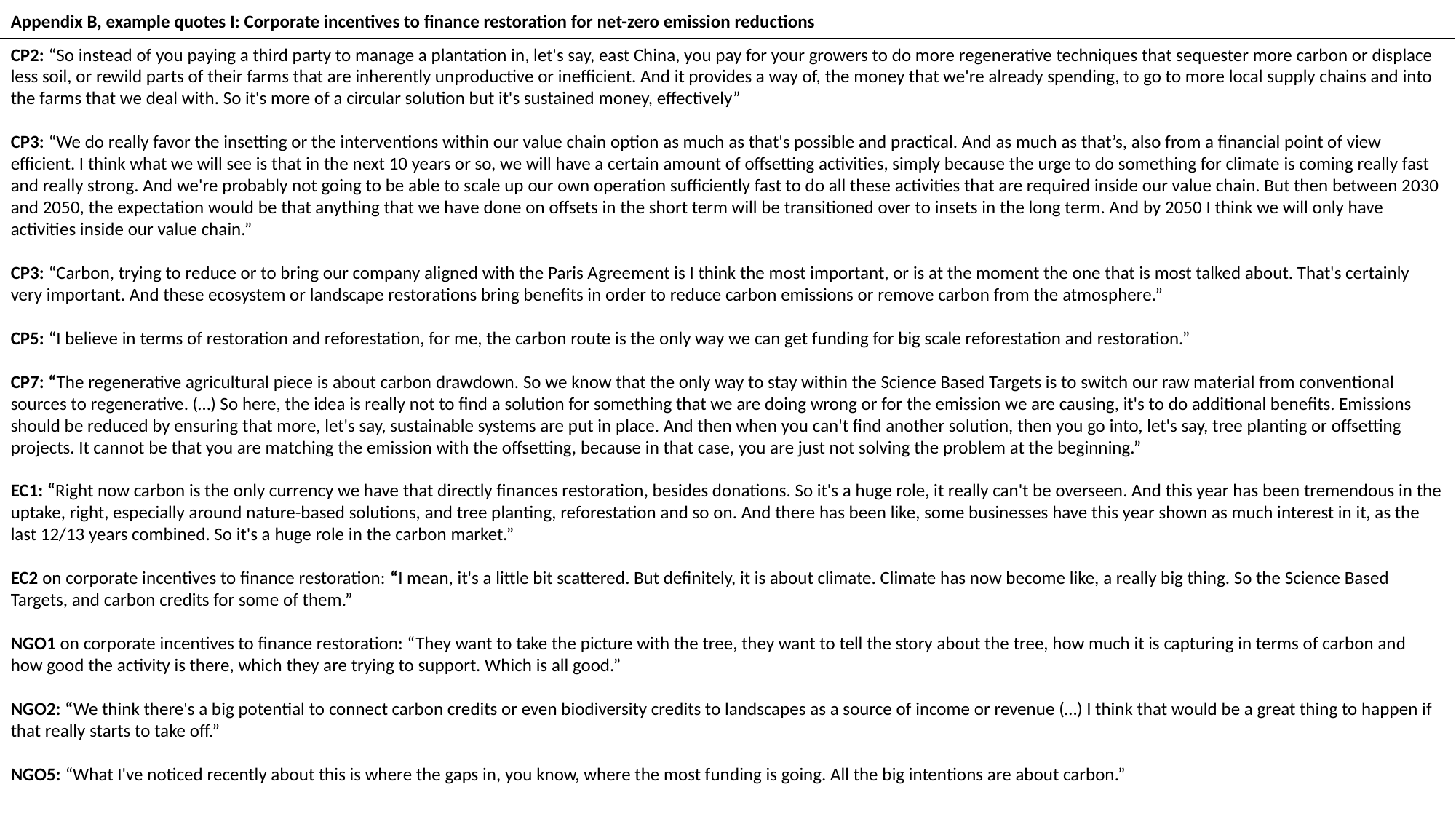

Appendix B, example quotes I: Corporate incentives to finance restoration for net-zero emission reductions
CP2: “So instead of you paying a third party to manage a plantation in, let's say, east China, you pay for your growers to do more regenerative techniques that sequester more carbon or displace less soil, or rewild parts of their farms that are inherently unproductive or inefficient. And it provides a way of, the money that we're already spending, to go to more local supply chains and into the farms that we deal with. So it's more of a circular solution but it's sustained money, effectively”
CP3: “We do really favor the insetting or the interventions within our value chain option as much as that's possible and practical. And as much as that’s, also from a financial point of view efficient. I think what we will see is that in the next 10 years or so, we will have a certain amount of offsetting activities, simply because the urge to do something for climate is coming really fast and really strong. And we're probably not going to be able to scale up our own operation sufficiently fast to do all these activities that are required inside our value chain. But then between 2030 and 2050, the expectation would be that anything that we have done on offsets in the short term will be transitioned over to insets in the long term. And by 2050 I think we will only have activities inside our value chain.”
CP3: “Carbon, trying to reduce or to bring our company aligned with the Paris Agreement is I think the most important, or is at the moment the one that is most talked about. That's certainly very important. And these ecosystem or landscape restorations bring benefits in order to reduce carbon emissions or remove carbon from the atmosphere.”
CP5: “I believe in terms of restoration and reforestation, for me, the carbon route is the only way we can get funding for big scale reforestation and restoration.”
CP7: “The regenerative agricultural piece is about carbon drawdown. So we know that the only way to stay within the Science Based Targets is to switch our raw material from conventional sources to regenerative. (…) So here, the idea is really not to find a solution for something that we are doing wrong or for the emission we are causing, it's to do additional benefits. Emissions should be reduced by ensuring that more, let's say, sustainable systems are put in place. And then when you can't find another solution, then you go into, let's say, tree planting or offsetting projects. It cannot be that you are matching the emission with the offsetting, because in that case, you are just not solving the problem at the beginning.”
EC1: “Right now carbon is the only currency we have that directly finances restoration, besides donations. So it's a huge role, it really can't be overseen. And this year has been tremendous in the uptake, right, especially around nature-based solutions, and tree planting, reforestation and so on. And there has been like, some businesses have this year shown as much interest in it, as the last 12/13 years combined. So it's a huge role in the carbon market.”
EC2 on corporate incentives to finance restoration: “I mean, it's a little bit scattered. But definitely, it is about climate. Climate has now become like, a really big thing. So the Science Based Targets, and carbon credits for some of them.”
NGO1 on corporate incentives to finance restoration: “They want to take the picture with the tree, they want to tell the story about the tree, how much it is capturing in terms of carbon and how good the activity is there, which they are trying to support. Which is all good.”
NGO2: “We think there's a big potential to connect carbon credits or even biodiversity credits to landscapes as a source of income or revenue (…) I think that would be a great thing to happen if that really starts to take off.”
NGO5: “What I've noticed recently about this is where the gaps in, you know, where the most funding is going. All the big intentions are about carbon.”

## Slide 4
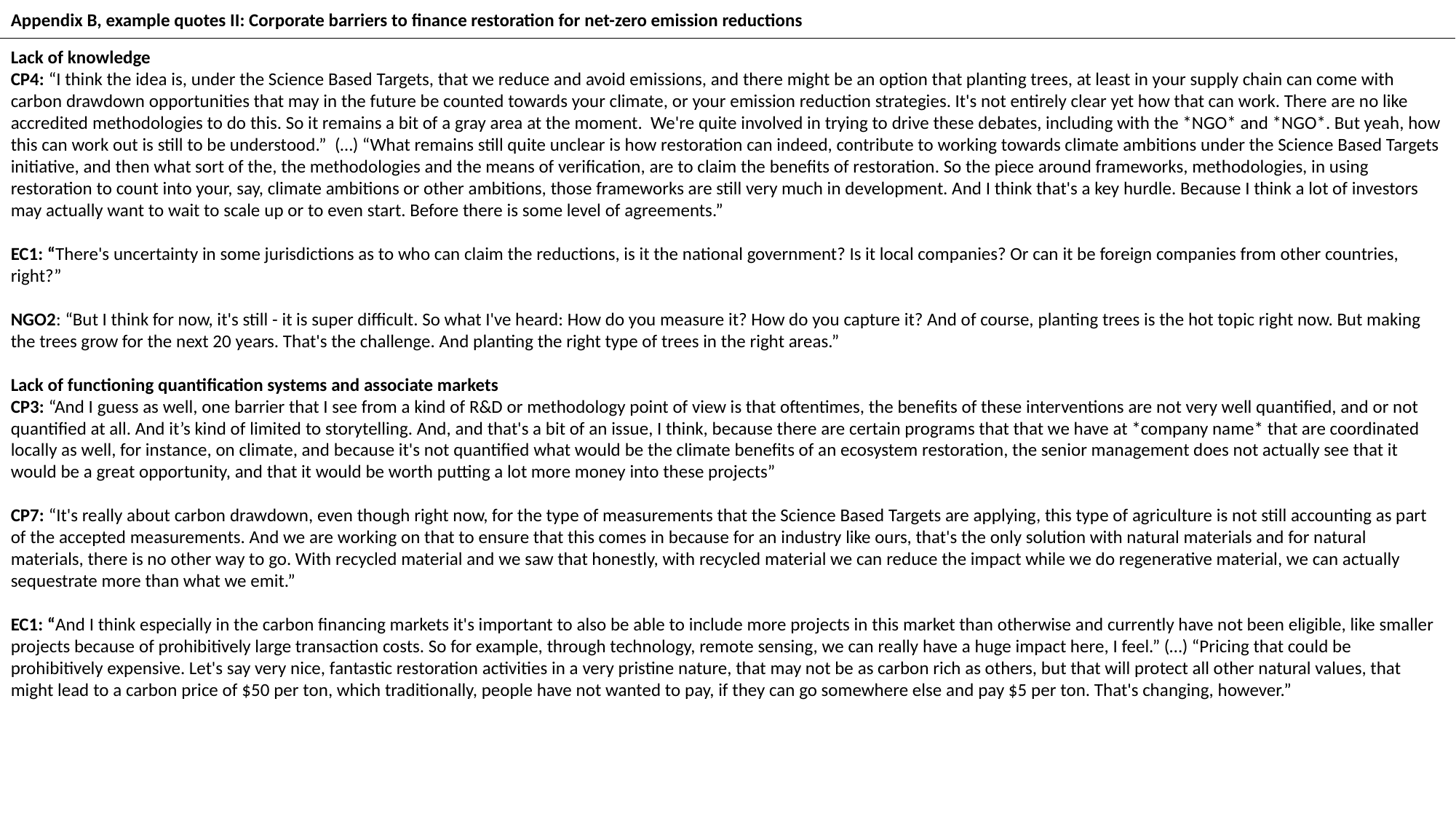

Appendix B, example quotes II: Corporate barriers to finance restoration for net-zero emission reductions
Lack of knowledge
CP4: “I think the idea is, under the Science Based Targets, that we reduce and avoid emissions, and there might be an option that planting trees, at least in your supply chain can come with carbon drawdown opportunities that may in the future be counted towards your climate, or your emission reduction strategies. It's not entirely clear yet how that can work. There are no like accredited methodologies to do this. So it remains a bit of a gray area at the moment. We're quite involved in trying to drive these debates, including with the *NGO* and *NGO*. But yeah, how this can work out is still to be understood.” (…) “What remains still quite unclear is how restoration can indeed, contribute to working towards climate ambitions under the Science Based Targets initiative, and then what sort of the, the methodologies and the means of verification, are to claim the benefits of restoration. So the piece around frameworks, methodologies, in using restoration to count into your, say, climate ambitions or other ambitions, those frameworks are still very much in development. And I think that's a key hurdle. Because I think a lot of investors may actually want to wait to scale up or to even start. Before there is some level of agreements.”
EC1: “There's uncertainty in some jurisdictions as to who can claim the reductions, is it the national government? Is it local companies? Or can it be foreign companies from other countries, right?”
NGO2: “But I think for now, it's still - it is super difficult. So what I've heard: How do you measure it? How do you capture it? And of course, planting trees is the hot topic right now. But making the trees grow for the next 20 years. That's the challenge. And planting the right type of trees in the right areas.”
Lack of functioning quantification systems and associate markets
CP3: “And I guess as well, one barrier that I see from a kind of R&D or methodology point of view is that oftentimes, the benefits of these interventions are not very well quantified, and or not quantified at all. And it’s kind of limited to storytelling. And, and that's a bit of an issue, I think, because there are certain programs that that we have at *company name* that are coordinated locally as well, for instance, on climate, and because it's not quantified what would be the climate benefits of an ecosystem restoration, the senior management does not actually see that it would be a great opportunity, and that it would be worth putting a lot more money into these projects”
CP7: “It's really about carbon drawdown, even though right now, for the type of measurements that the Science Based Targets are applying, this type of agriculture is not still accounting as part of the accepted measurements. And we are working on that to ensure that this comes in because for an industry like ours, that's the only solution with natural materials and for natural materials, there is no other way to go. With recycled material and we saw that honestly, with recycled material we can reduce the impact while we do regenerative material, we can actually sequestrate more than what we emit.”
EC1: “And I think especially in the carbon financing markets it's important to also be able to include more projects in this market than otherwise and currently have not been eligible, like smaller projects because of prohibitively large transaction costs. So for example, through technology, remote sensing, we can really have a huge impact here, I feel.” (…) “Pricing that could be prohibitively expensive. Let's say very nice, fantastic restoration activities in a very pristine nature, that may not be as carbon rich as others, but that will protect all other natural values, that might lead to a carbon price of $50 per ton, which traditionally, people have not wanted to pay, if they can go somewhere else and pay $5 per ton. That's changing, however.”

## Slide 5
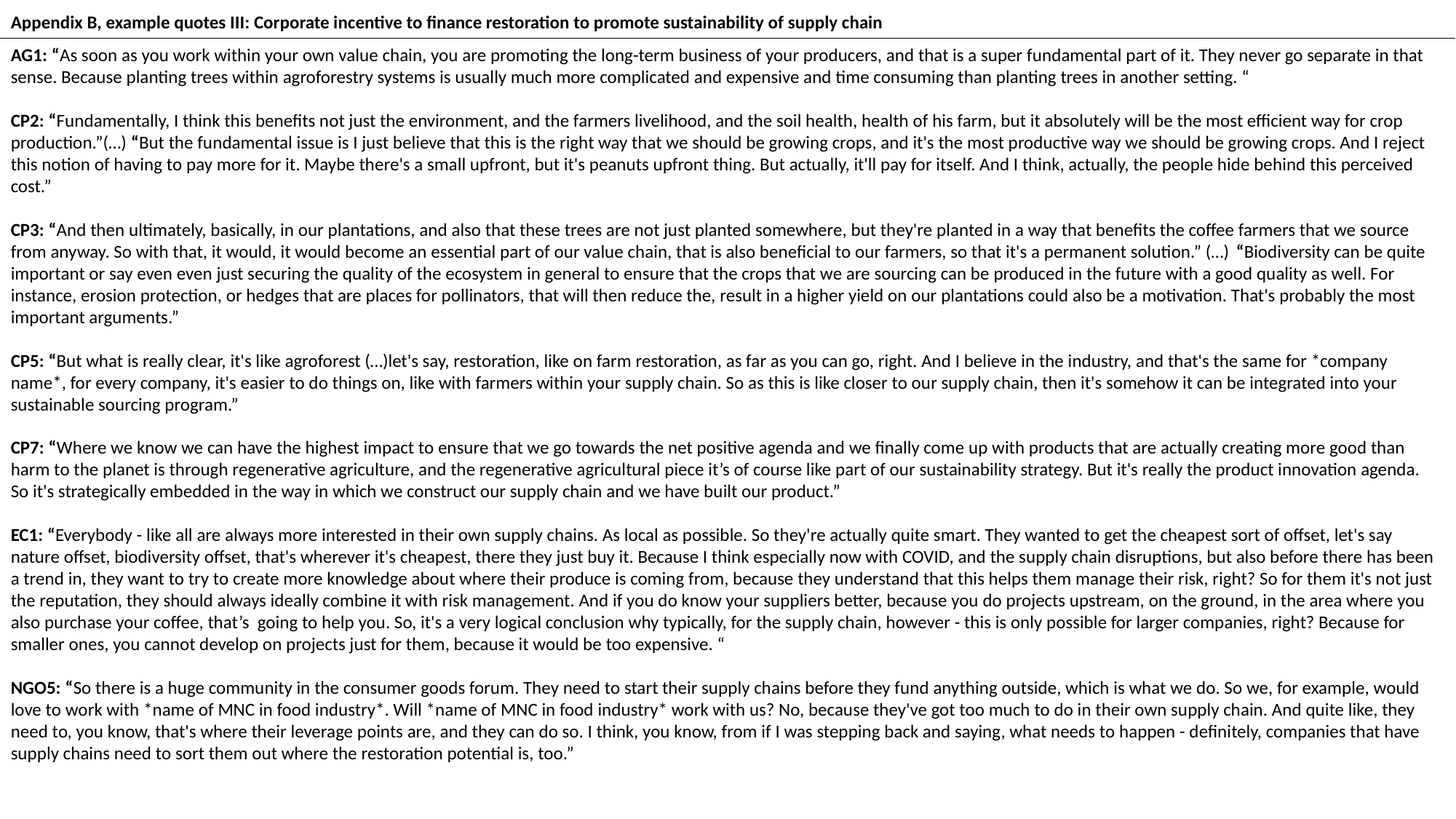

Appendix B, example quotes III: Corporate incentive to finance restoration to promote sustainability of supply chain
AG1: “As soon as you work within your own value chain, you are promoting the long-term business of your producers, and that is a super fundamental part of it. They never go separate in that sense. Because planting trees within agroforestry systems is usually much more complicated and expensive and time consuming than planting trees in another setting. “
CP2: “Fundamentally, I think this benefits not just the environment, and the farmers livelihood, and the soil health, health of his farm, but it absolutely will be the most efficient way for crop production.”(…) “But the fundamental issue is I just believe that this is the right way that we should be growing crops, and it's the most productive way we should be growing crops. And I reject this notion of having to pay more for it. Maybe there's a small upfront, but it's peanuts upfront thing. But actually, it'll pay for itself. And I think, actually, the people hide behind this perceived cost.”
CP3: “And then ultimately, basically, in our plantations, and also that these trees are not just planted somewhere, but they're planted in a way that benefits the coffee farmers that we source from anyway. So with that, it would, it would become an essential part of our value chain, that is also beneficial to our farmers, so that it's a permanent solution.” (…) “Biodiversity can be quite important or say even even just securing the quality of the ecosystem in general to ensure that the crops that we are sourcing can be produced in the future with a good quality as well. For instance, erosion protection, or hedges that are places for pollinators, that will then reduce the, result in a higher yield on our plantations could also be a motivation. That's probably the most important arguments.”
CP5: “But what is really clear, it's like agroforest (…)let's say, restoration, like on farm restoration, as far as you can go, right. And I believe in the industry, and that's the same for *company name*, for every company, it's easier to do things on, like with farmers within your supply chain. So as this is like closer to our supply chain, then it's somehow it can be integrated into your sustainable sourcing program.”
CP7: “Where we know we can have the highest impact to ensure that we go towards the net positive agenda and we finally come up with products that are actually creating more good than harm to the planet is through regenerative agriculture, and the regenerative agricultural piece it’s of course like part of our sustainability strategy. But it's really the product innovation agenda. So it's strategically embedded in the way in which we construct our supply chain and we have built our product.”
EC1: “Everybody - like all are always more interested in their own supply chains. As local as possible. So they're actually quite smart. They wanted to get the cheapest sort of offset, let's say nature offset, biodiversity offset, that's wherever it's cheapest, there they just buy it. Because I think especially now with COVID, and the supply chain disruptions, but also before there has been a trend in, they want to try to create more knowledge about where their produce is coming from, because they understand that this helps them manage their risk, right? So for them it's not just the reputation, they should always ideally combine it with risk management. And if you do know your suppliers better, because you do projects upstream, on the ground, in the area where you also purchase your coffee, that’s going to help you. So, it's a very logical conclusion why typically, for the supply chain, however - this is only possible for larger companies, right? Because for smaller ones, you cannot develop on projects just for them, because it would be too expensive. “
NGO5: “So there is a huge community in the consumer goods forum. They need to start their supply chains before they fund anything outside, which is what we do. So we, for example, would love to work with *name of MNC in food industry*. Will *name of MNC in food industry* work with us? No, because they've got too much to do in their own supply chain. And quite like, they need to, you know, that's where their leverage points are, and they can do so. I think, you know, from if I was stepping back and saying, what needs to happen - definitely, companies that have supply chains need to sort them out where the restoration potential is, too.”

## Slide 6
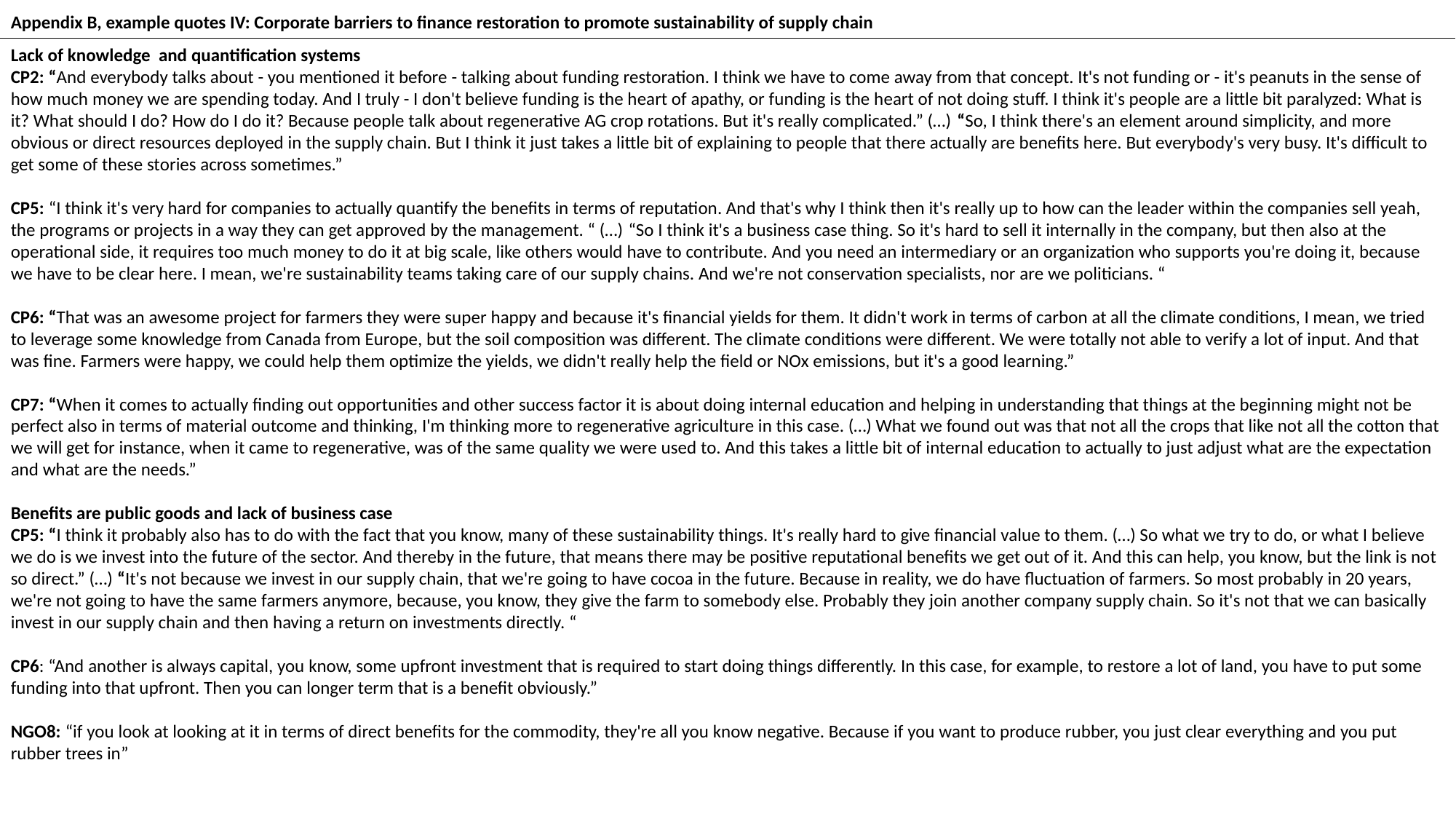

Appendix B, example quotes IV: Corporate barriers to finance restoration to promote sustainability of supply chain
Lack of knowledge and quantification systems
CP2: “And everybody talks about - you mentioned it before - talking about funding restoration. I think we have to come away from that concept. It's not funding or - it's peanuts in the sense of how much money we are spending today. And I truly - I don't believe funding is the heart of apathy, or funding is the heart of not doing stuff. I think it's people are a little bit paralyzed: What is it? What should I do? How do I do it? Because people talk about regenerative AG crop rotations. But it's really complicated.” (…) “So, I think there's an element around simplicity, and more obvious or direct resources deployed in the supply chain. But I think it just takes a little bit of explaining to people that there actually are benefits here. But everybody's very busy. It's difficult to get some of these stories across sometimes.”
CP5: “I think it's very hard for companies to actually quantify the benefits in terms of reputation. And that's why I think then it's really up to how can the leader within the companies sell yeah, the programs or projects in a way they can get approved by the management. “ (…) “So I think it's a business case thing. So it's hard to sell it internally in the company, but then also at the operational side, it requires too much money to do it at big scale, like others would have to contribute. And you need an intermediary or an organization who supports you're doing it, because we have to be clear here. I mean, we're sustainability teams taking care of our supply chains. And we're not conservation specialists, nor are we politicians. “
CP6: “That was an awesome project for farmers they were super happy and because it's financial yields for them. It didn't work in terms of carbon at all the climate conditions, I mean, we tried to leverage some knowledge from Canada from Europe, but the soil composition was different. The climate conditions were different. We were totally not able to verify a lot of input. And that was fine. Farmers were happy, we could help them optimize the yields, we didn't really help the field or NOx emissions, but it's a good learning.”
CP7: “When it comes to actually finding out opportunities and other success factor it is about doing internal education and helping in understanding that things at the beginning might not be perfect also in terms of material outcome and thinking, I'm thinking more to regenerative agriculture in this case. (…) What we found out was that not all the crops that like not all the cotton that we will get for instance, when it came to regenerative, was of the same quality we were used to. And this takes a little bit of internal education to actually to just adjust what are the expectation and what are the needs.”
Benefits are public goods and lack of business case
CP5: “I think it probably also has to do with the fact that you know, many of these sustainability things. It's really hard to give financial value to them. (…) So what we try to do, or what I believe we do is we invest into the future of the sector. And thereby in the future, that means there may be positive reputational benefits we get out of it. And this can help, you know, but the link is not so direct.” (…) “It's not because we invest in our supply chain, that we're going to have cocoa in the future. Because in reality, we do have fluctuation of farmers. So most probably in 20 years, we're not going to have the same farmers anymore, because, you know, they give the farm to somebody else. Probably they join another company supply chain. So it's not that we can basically invest in our supply chain and then having a return on investments directly. “
CP6: “And another is always capital, you know, some upfront investment that is required to start doing things differently. In this case, for example, to restore a lot of land, you have to put some funding into that upfront. Then you can longer term that is a benefit obviously.”
NGO8: “if you look at looking at it in terms of direct benefits for the commodity, they're all you know negative. Because if you want to produce rubber, you just clear everything and you put rubber trees in”

## Slide 7
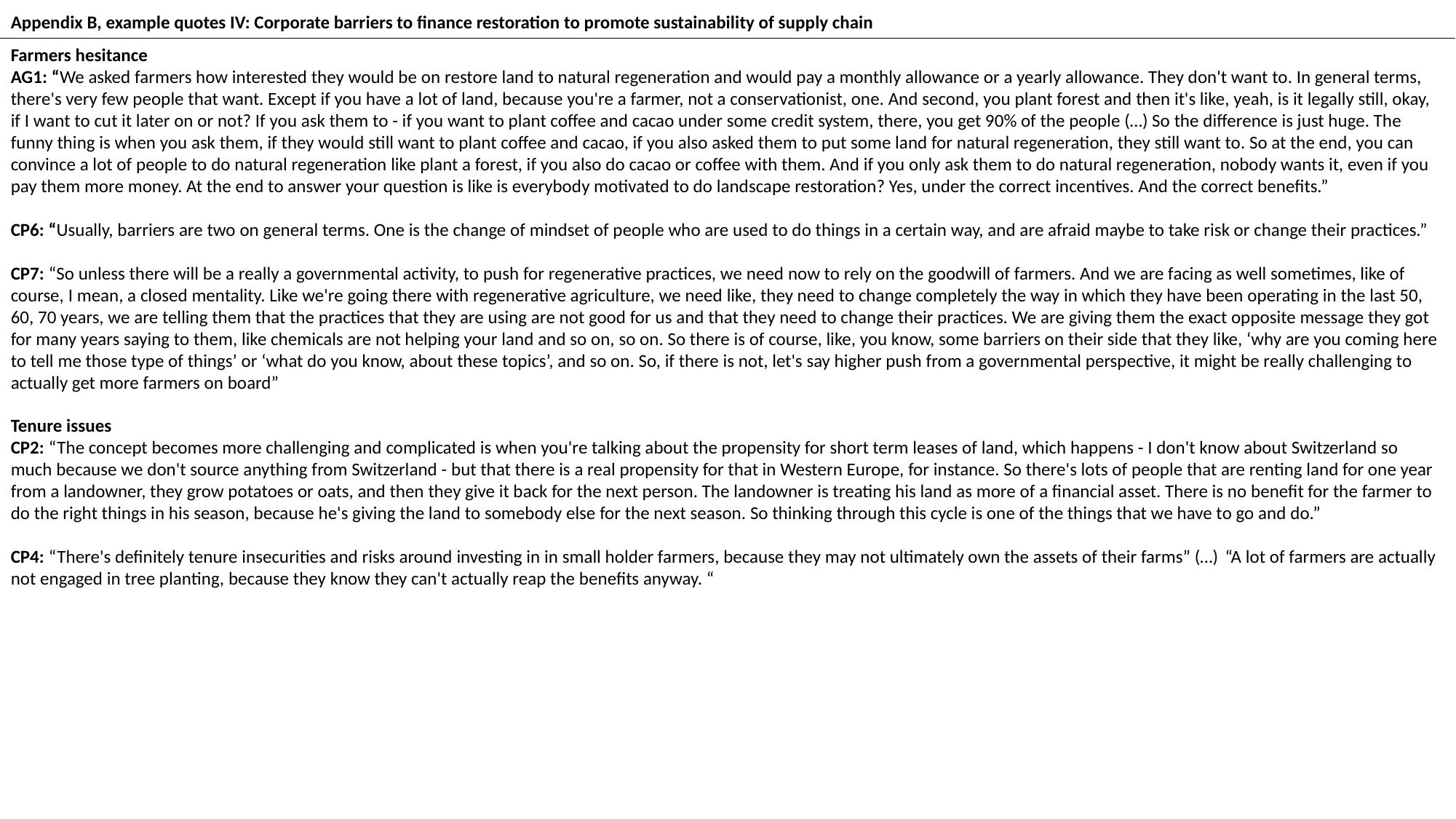

Appendix B, example quotes IV: Corporate barriers to finance restoration to promote sustainability of supply chain
Farmers hesitance
AG1: “We asked farmers how interested they would be on restore land to natural regeneration and would pay a monthly allowance or a yearly allowance. They don't want to. In general terms, there's very few people that want. Except if you have a lot of land, because you're a farmer, not a conservationist, one. And second, you plant forest and then it's like, yeah, is it legally still, okay, if I want to cut it later on or not? If you ask them to - if you want to plant coffee and cacao under some credit system, there, you get 90% of the people (…) So the difference is just huge. The funny thing is when you ask them, if they would still want to plant coffee and cacao, if you also asked them to put some land for natural regeneration, they still want to. So at the end, you can convince a lot of people to do natural regeneration like plant a forest, if you also do cacao or coffee with them. And if you only ask them to do natural regeneration, nobody wants it, even if you pay them more money. At the end to answer your question is like is everybody motivated to do landscape restoration? Yes, under the correct incentives. And the correct benefits.”
CP6: “Usually, barriers are two on general terms. One is the change of mindset of people who are used to do things in a certain way, and are afraid maybe to take risk or change their practices.”
CP7: “So unless there will be a really a governmental activity, to push for regenerative practices, we need now to rely on the goodwill of farmers. And we are facing as well sometimes, like of course, I mean, a closed mentality. Like we're going there with regenerative agriculture, we need like, they need to change completely the way in which they have been operating in the last 50, 60, 70 years, we are telling them that the practices that they are using are not good for us and that they need to change their practices. We are giving them the exact opposite message they got for many years saying to them, like chemicals are not helping your land and so on, so on. So there is of course, like, you know, some barriers on their side that they like, ‘why are you coming here to tell me those type of things’ or ‘what do you know, about these topics’, and so on. So, if there is not, let's say higher push from a governmental perspective, it might be really challenging to actually get more farmers on board”
Tenure issues
CP2: “The concept becomes more challenging and complicated is when you're talking about the propensity for short term leases of land, which happens - I don't know about Switzerland so much because we don't source anything from Switzerland - but that there is a real propensity for that in Western Europe, for instance. So there's lots of people that are renting land for one year from a landowner, they grow potatoes or oats, and then they give it back for the next person. The landowner is treating his land as more of a financial asset. There is no benefit for the farmer to do the right things in his season, because he's giving the land to somebody else for the next season. So thinking through this cycle is one of the things that we have to go and do.”
CP4: “There's definitely tenure insecurities and risks around investing in in small holder farmers, because they may not ultimately own the assets of their farms” (…) “A lot of farmers are actually not engaged in tree planting, because they know they can't actually reap the benefits anyway. “

## Slide 8
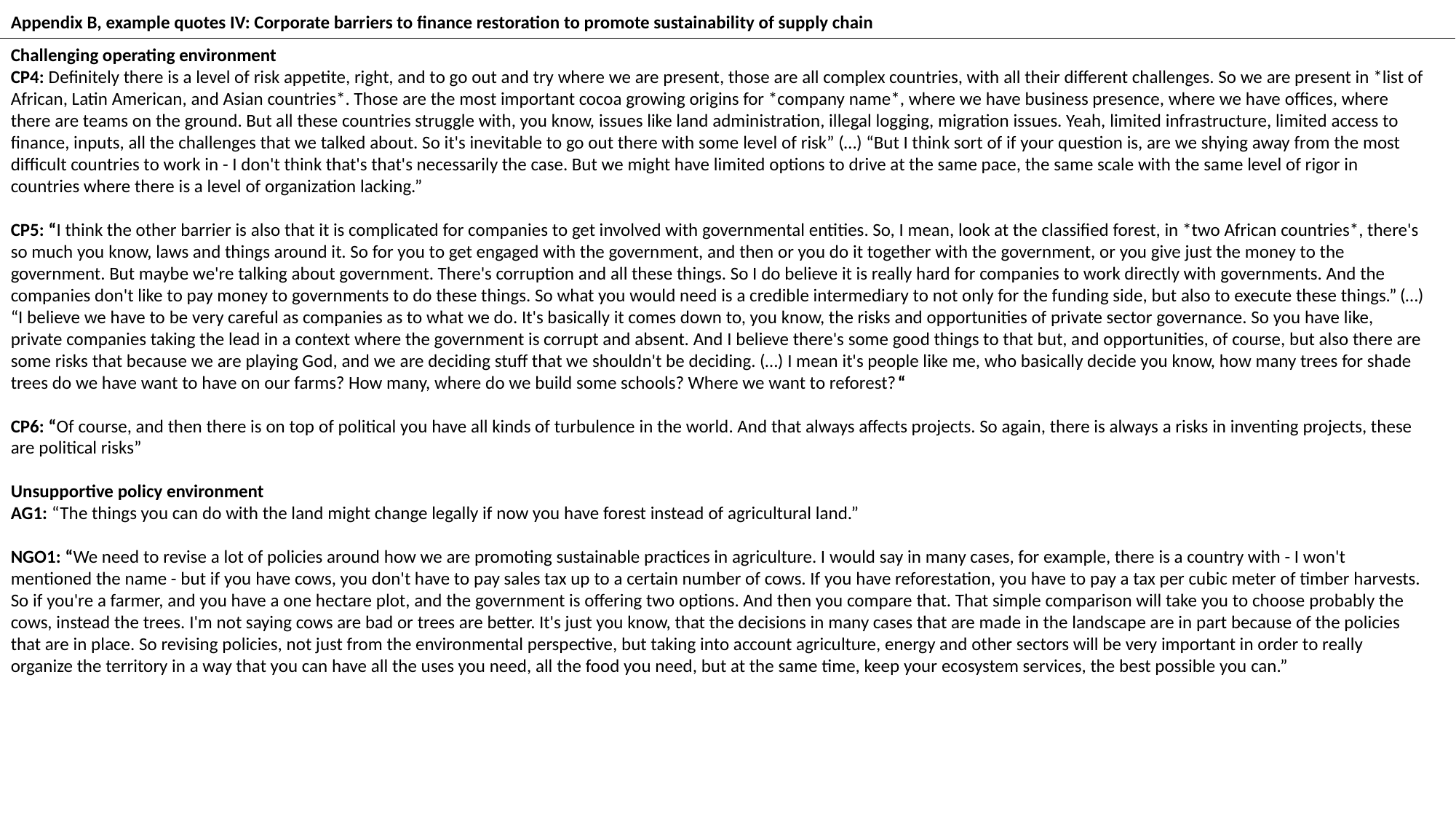

Appendix B, example quotes IV: Corporate barriers to finance restoration to promote sustainability of supply chain
Challenging operating environment
CP4: Definitely there is a level of risk appetite, right, and to go out and try where we are present, those are all complex countries, with all their different challenges. So we are present in *list of African, Latin American, and Asian countries*. Those are the most important cocoa growing origins for *company name*, where we have business presence, where we have offices, where there are teams on the ground. But all these countries struggle with, you know, issues like land administration, illegal logging, migration issues. Yeah, limited infrastructure, limited access to finance, inputs, all the challenges that we talked about. So it's inevitable to go out there with some level of risk” (…) “But I think sort of if your question is, are we shying away from the most difficult countries to work in - I don't think that's that's necessarily the case. But we might have limited options to drive at the same pace, the same scale with the same level of rigor in countries where there is a level of organization lacking.”
CP5: “I think the other barrier is also that it is complicated for companies to get involved with governmental entities. So, I mean, look at the classified forest, in *two African countries*, there's so much you know, laws and things around it. So for you to get engaged with the government, and then or you do it together with the government, or you give just the money to the government. But maybe we're talking about government. There's corruption and all these things. So I do believe it is really hard for companies to work directly with governments. And the companies don't like to pay money to governments to do these things. So what you would need is a credible intermediary to not only for the funding side, but also to execute these things.”(…) “I believe we have to be very careful as companies as to what we do. It's basically it comes down to, you know, the risks and opportunities of private sector governance. So you have like, private companies taking the lead in a context where the government is corrupt and absent. And I believe there's some good things to that but, and opportunities, of course, but also there are some risks that because we are playing God, and we are deciding stuff that we shouldn't be deciding. (…) I mean it's people like me, who basically decide you know, how many trees for shade trees do we have want to have on our farms? How many, where do we build some schools? Where we want to reforest?“
CP6: “Of course, and then there is on top of political you have all kinds of turbulence in the world. And that always affects projects. So again, there is always a risks in inventing projects, these are political risks”
Unsupportive policy environment
AG1: “The things you can do with the land might change legally if now you have forest instead of agricultural land.”
NGO1: “We need to revise a lot of policies around how we are promoting sustainable practices in agriculture. I would say in many cases, for example, there is a country with - I won't mentioned the name - but if you have cows, you don't have to pay sales tax up to a certain number of cows. If you have reforestation, you have to pay a tax per cubic meter of timber harvests. So if you're a farmer, and you have a one hectare plot, and the government is offering two options. And then you compare that. That simple comparison will take you to choose probably the cows, instead the trees. I'm not saying cows are bad or trees are better. It's just you know, that the decisions in many cases that are made in the landscape are in part because of the policies that are in place. So revising policies, not just from the environmental perspective, but taking into account agriculture, energy and other sectors will be very important in order to really organize the territory in a way that you can have all the uses you need, all the food you need, but at the same time, keep your ecosystem services, the best possible you can.”

## Slide 9
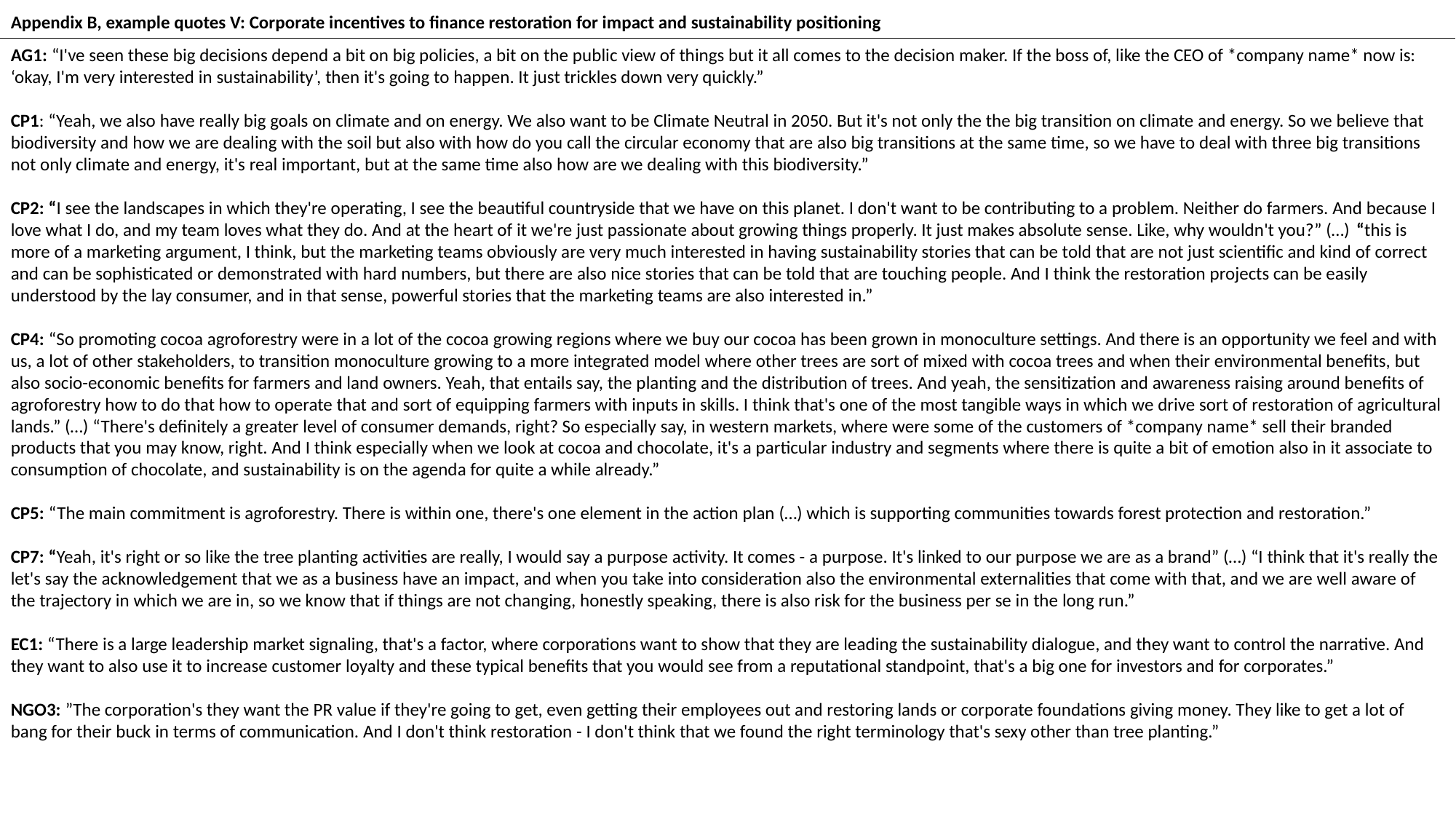

Appendix B, example quotes V: Corporate incentives to finance restoration for impact and sustainability positioning
AG1: “I've seen these big decisions depend a bit on big policies, a bit on the public view of things but it all comes to the decision maker. If the boss of, like the CEO of *company name* now is: ‘okay, I'm very interested in sustainability’, then it's going to happen. It just trickles down very quickly.”
CP1: “Yeah, we also have really big goals on climate and on energy. We also want to be Climate Neutral in 2050. But it's not only the the big transition on climate and energy. So we believe that biodiversity and how we are dealing with the soil but also with how do you call the circular economy that are also big transitions at the same time, so we have to deal with three big transitions not only climate and energy, it's real important, but at the same time also how are we dealing with this biodiversity.”
CP2: “I see the landscapes in which they're operating, I see the beautiful countryside that we have on this planet. I don't want to be contributing to a problem. Neither do farmers. And because I love what I do, and my team loves what they do. And at the heart of it we're just passionate about growing things properly. It just makes absolute sense. Like, why wouldn't you?” (…) “this is more of a marketing argument, I think, but the marketing teams obviously are very much interested in having sustainability stories that can be told that are not just scientific and kind of correct and can be sophisticated or demonstrated with hard numbers, but there are also nice stories that can be told that are touching people. And I think the restoration projects can be easily understood by the lay consumer, and in that sense, powerful stories that the marketing teams are also interested in.”
CP4: “So promoting cocoa agroforestry were in a lot of the cocoa growing regions where we buy our cocoa has been grown in monoculture settings. And there is an opportunity we feel and with us, a lot of other stakeholders, to transition monoculture growing to a more integrated model where other trees are sort of mixed with cocoa trees and when their environmental benefits, but also socio-economic benefits for farmers and land owners. Yeah, that entails say, the planting and the distribution of trees. And yeah, the sensitization and awareness raising around benefits of agroforestry how to do that how to operate that and sort of equipping farmers with inputs in skills. I think that's one of the most tangible ways in which we drive sort of restoration of agricultural lands.” (…) “There's definitely a greater level of consumer demands, right? So especially say, in western markets, where were some of the customers of *company name* sell their branded products that you may know, right. And I think especially when we look at cocoa and chocolate, it's a particular industry and segments where there is quite a bit of emotion also in it associate to consumption of chocolate, and sustainability is on the agenda for quite a while already.”
CP5: “The main commitment is agroforestry. There is within one, there's one element in the action plan (…) which is supporting communities towards forest protection and restoration.”
CP7: “Yeah, it's right or so like the tree planting activities are really, I would say a purpose activity. It comes - a purpose. It's linked to our purpose we are as a brand” (…) “I think that it's really the let's say the acknowledgement that we as a business have an impact, and when you take into consideration also the environmental externalities that come with that, and we are well aware of the trajectory in which we are in, so we know that if things are not changing, honestly speaking, there is also risk for the business per se in the long run.”
EC1: “There is a large leadership market signaling, that's a factor, where corporations want to show that they are leading the sustainability dialogue, and they want to control the narrative. And they want to also use it to increase customer loyalty and these typical benefits that you would see from a reputational standpoint, that's a big one for investors and for corporates.”
NGO3: ”The corporation's they want the PR value if they're going to get, even getting their employees out and restoring lands or corporate foundations giving money. They like to get a lot of bang for their buck in terms of communication. And I don't think restoration - I don't think that we found the right terminology that's sexy other than tree planting.”

## Slide 10
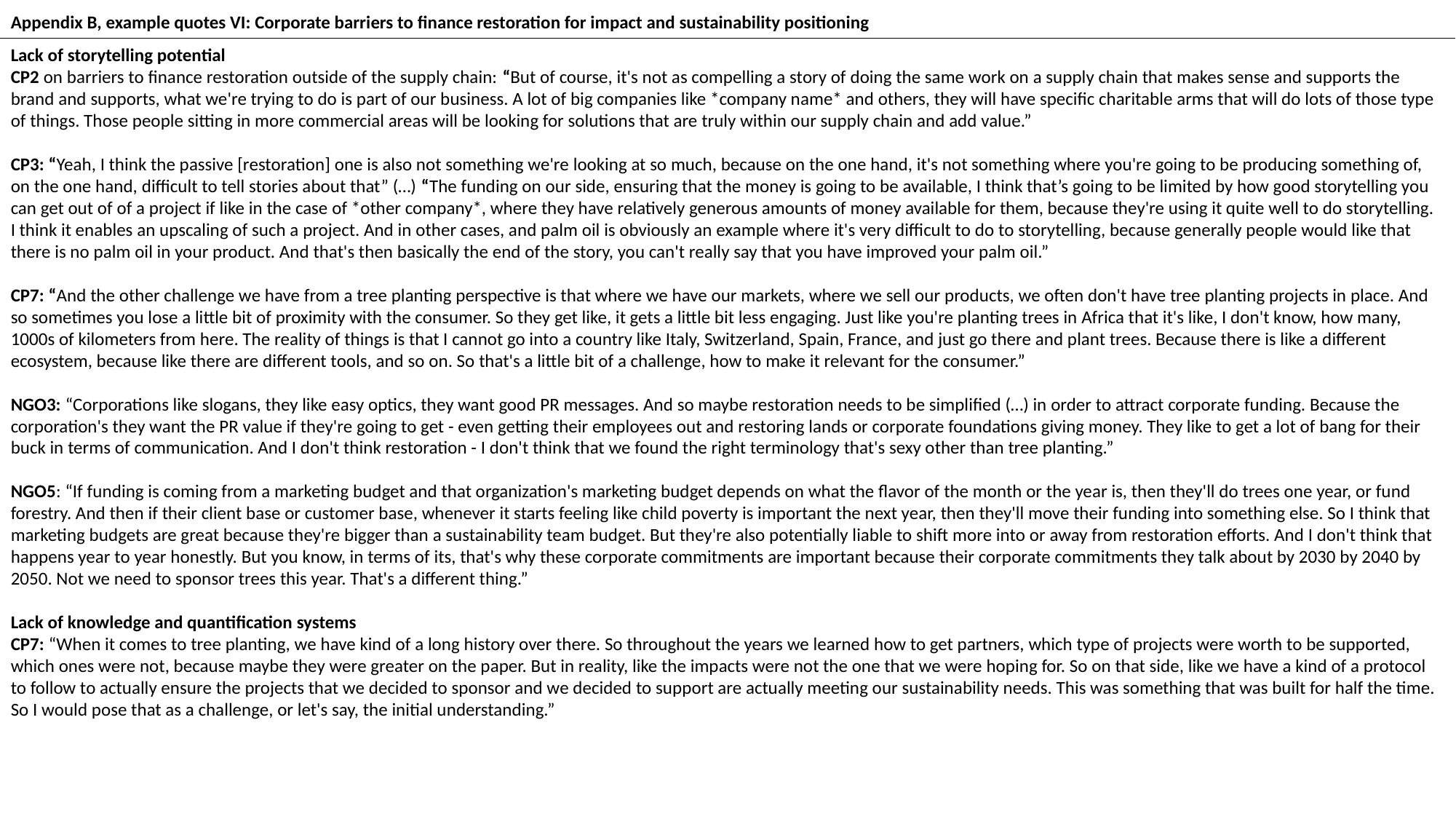

Appendix B, example quotes VI: Corporate barriers to finance restoration for impact and sustainability positioning
Lack of storytelling potential
CP2 on barriers to finance restoration outside of the supply chain: “But of course, it's not as compelling a story of doing the same work on a supply chain that makes sense and supports the brand and supports, what we're trying to do is part of our business. A lot of big companies like *company name* and others, they will have specific charitable arms that will do lots of those type of things. Those people sitting in more commercial areas will be looking for solutions that are truly within our supply chain and add value.”
CP3: “Yeah, I think the passive [restoration] one is also not something we're looking at so much, because on the one hand, it's not something where you're going to be producing something of, on the one hand, difficult to tell stories about that” (…) “The funding on our side, ensuring that the money is going to be available, I think that’s going to be limited by how good storytelling you can get out of of a project if like in the case of *other company*, where they have relatively generous amounts of money available for them, because they're using it quite well to do storytelling. I think it enables an upscaling of such a project. And in other cases, and palm oil is obviously an example where it's very difficult to do to storytelling, because generally people would like that there is no palm oil in your product. And that's then basically the end of the story, you can't really say that you have improved your palm oil.”
CP7: “And the other challenge we have from a tree planting perspective is that where we have our markets, where we sell our products, we often don't have tree planting projects in place. And so sometimes you lose a little bit of proximity with the consumer. So they get like, it gets a little bit less engaging. Just like you're planting trees in Africa that it's like, I don't know, how many, 1000s of kilometers from here. The reality of things is that I cannot go into a country like Italy, Switzerland, Spain, France, and just go there and plant trees. Because there is like a different ecosystem, because like there are different tools, and so on. So that's a little bit of a challenge, how to make it relevant for the consumer.”
NGO3: “Corporations like slogans, they like easy optics, they want good PR messages. And so maybe restoration needs to be simplified (…) in order to attract corporate funding. Because the corporation's they want the PR value if they're going to get - even getting their employees out and restoring lands or corporate foundations giving money. They like to get a lot of bang for their buck in terms of communication. And I don't think restoration - I don't think that we found the right terminology that's sexy other than tree planting.”
NGO5: “If funding is coming from a marketing budget and that organization's marketing budget depends on what the flavor of the month or the year is, then they'll do trees one year, or fund forestry. And then if their client base or customer base, whenever it starts feeling like child poverty is important the next year, then they'll move their funding into something else. So I think that marketing budgets are great because they're bigger than a sustainability team budget. But they're also potentially liable to shift more into or away from restoration efforts. And I don't think that happens year to year honestly. But you know, in terms of its, that's why these corporate commitments are important because their corporate commitments they talk about by 2030 by 2040 by 2050. Not we need to sponsor trees this year. That's a different thing.”
Lack of knowledge and quantification systems
CP7: “When it comes to tree planting, we have kind of a long history over there. So throughout the years we learned how to get partners, which type of projects were worth to be supported, which ones were not, because maybe they were greater on the paper. But in reality, like the impacts were not the one that we were hoping for. So on that side, like we have a kind of a protocol to follow to actually ensure the projects that we decided to sponsor and we decided to support are actually meeting our sustainability needs. This was something that was built for half the time. So I would pose that as a challenge, or let's say, the initial understanding.”

## Slide 11
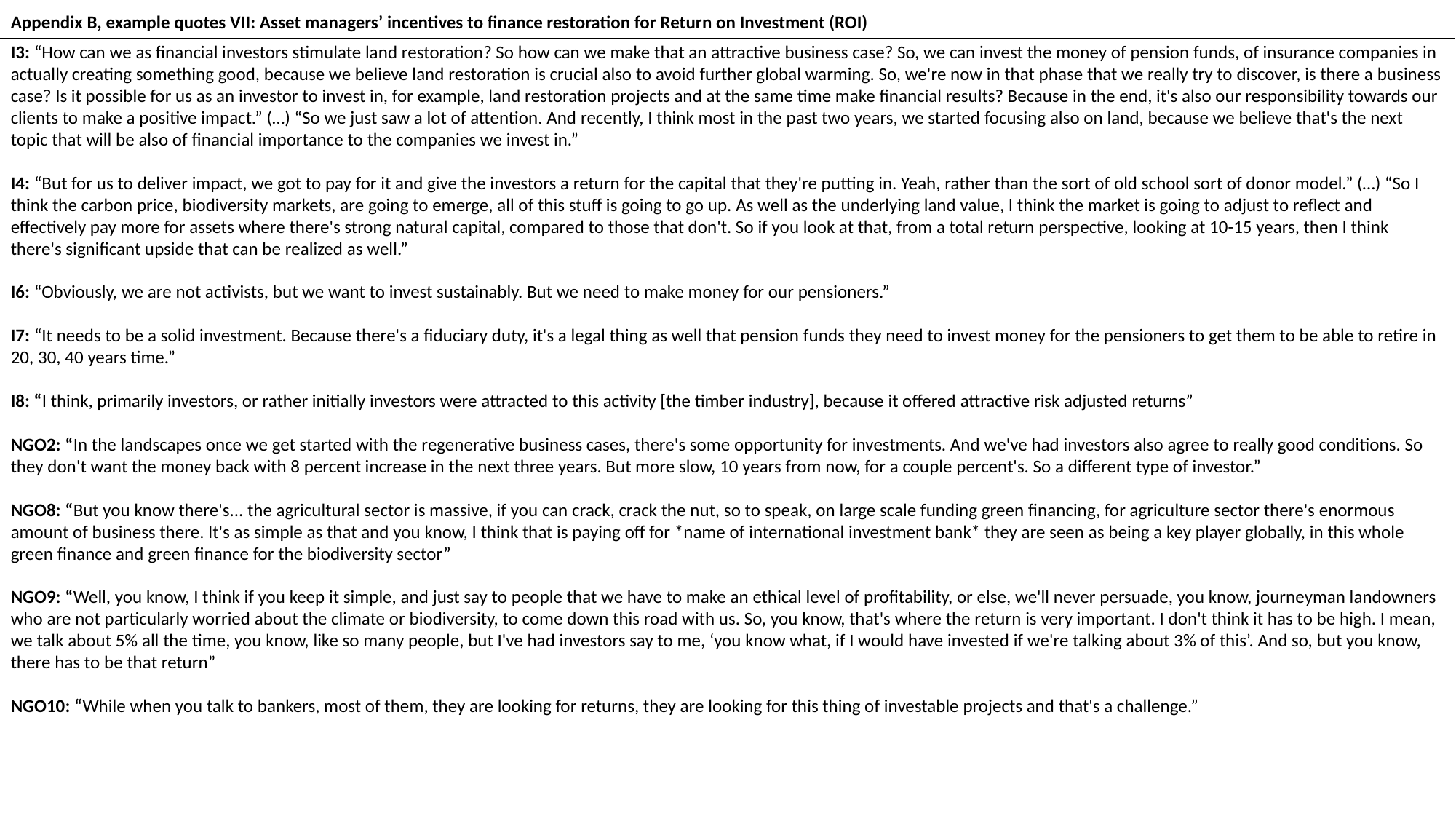

Appendix B, example quotes VII: Asset managers’ incentives to finance restoration for Return on Investment (ROI)
I3: “How can we as financial investors stimulate land restoration? So how can we make that an attractive business case? So, we can invest the money of pension funds, of insurance companies in actually creating something good, because we believe land restoration is crucial also to avoid further global warming. So, we're now in that phase that we really try to discover, is there a business case? Is it possible for us as an investor to invest in, for example, land restoration projects and at the same time make financial results? Because in the end, it's also our responsibility towards our clients to make a positive impact.” (…) “So we just saw a lot of attention. And recently, I think most in the past two years, we started focusing also on land, because we believe that's the next topic that will be also of financial importance to the companies we invest in.”
I4: “But for us to deliver impact, we got to pay for it and give the investors a return for the capital that they're putting in. Yeah, rather than the sort of old school sort of donor model.” (…) “So I think the carbon price, biodiversity markets, are going to emerge, all of this stuff is going to go up. As well as the underlying land value, I think the market is going to adjust to reflect and effectively pay more for assets where there's strong natural capital, compared to those that don't. So if you look at that, from a total return perspective, looking at 10-15 years, then I think there's significant upside that can be realized as well.”
I6: “Obviously, we are not activists, but we want to invest sustainably. But we need to make money for our pensioners.”
I7: “It needs to be a solid investment. Because there's a fiduciary duty, it's a legal thing as well that pension funds they need to invest money for the pensioners to get them to be able to retire in 20, 30, 40 years time.”
I8: “I think, primarily investors, or rather initially investors were attracted to this activity [the timber industry], because it offered attractive risk adjusted returns”
NGO2: “In the landscapes once we get started with the regenerative business cases, there's some opportunity for investments. And we've had investors also agree to really good conditions. So they don't want the money back with 8 percent increase in the next three years. But more slow, 10 years from now, for a couple percent's. So a different type of investor.”
NGO8: “But you know there's... the agricultural sector is massive, if you can crack, crack the nut, so to speak, on large scale funding green financing, for agriculture sector there's enormous amount of business there. It's as simple as that and you know, I think that is paying off for *name of international investment bank* they are seen as being a key player globally, in this whole green finance and green finance for the biodiversity sector”
NGO9: “Well, you know, I think if you keep it simple, and just say to people that we have to make an ethical level of profitability, or else, we'll never persuade, you know, journeyman landowners who are not particularly worried about the climate or biodiversity, to come down this road with us. So, you know, that's where the return is very important. I don't think it has to be high. I mean, we talk about 5% all the time, you know, like so many people, but I've had investors say to me, ‘you know what, if I would have invested if we're talking about 3% of this’. And so, but you know, there has to be that return”
NGO10: “While when you talk to bankers, most of them, they are looking for returns, they are looking for this thing of investable projects and that's a challenge.”

## Slide 12
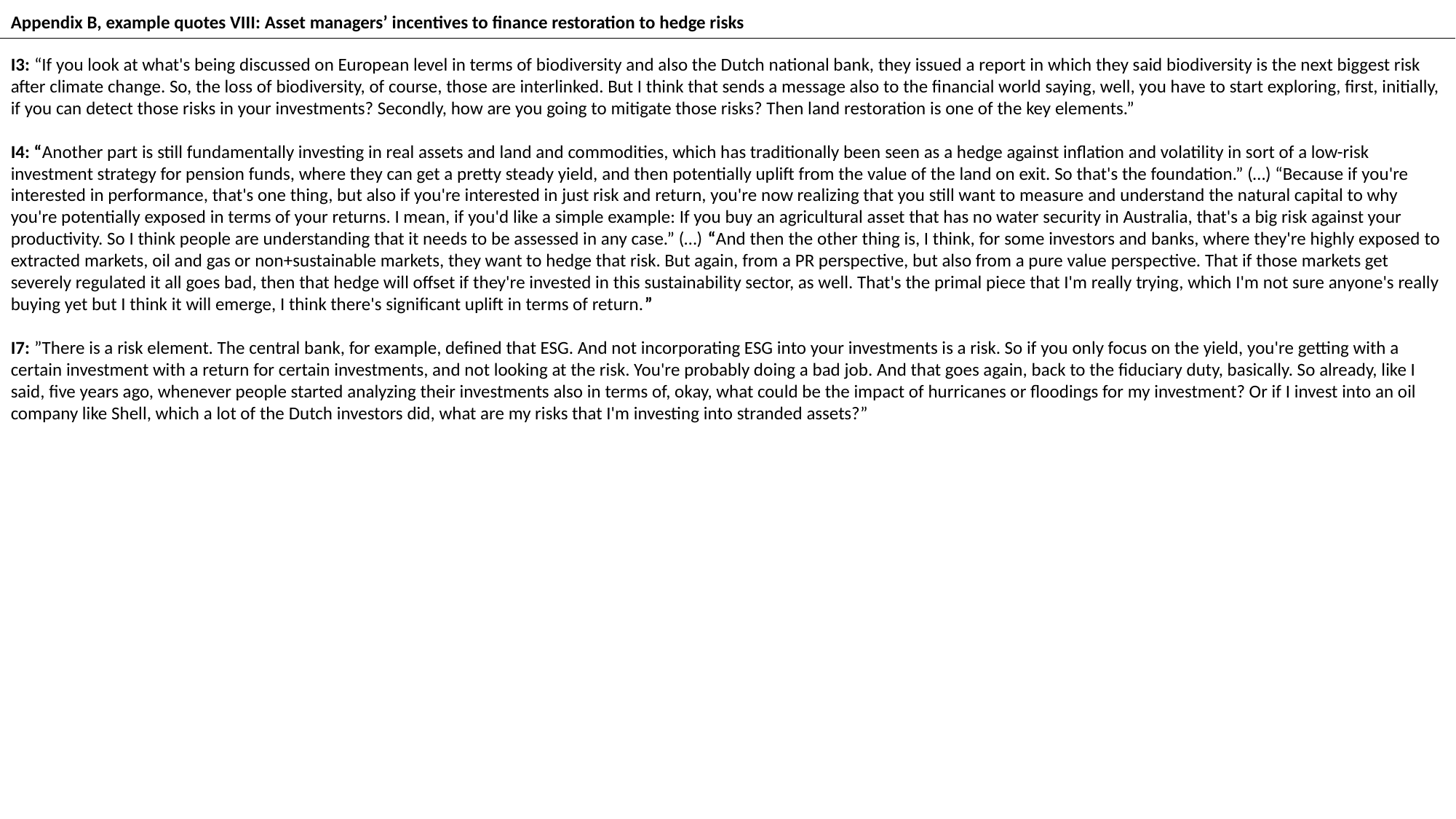

Appendix B, example quotes VIII: Asset managers’ incentives to finance restoration to hedge risks
I3: “If you look at what's being discussed on European level in terms of biodiversity and also the Dutch national bank, they issued a report in which they said biodiversity is the next biggest risk after climate change. So, the loss of biodiversity, of course, those are interlinked. But I think that sends a message also to the financial world saying, well, you have to start exploring, first, initially, if you can detect those risks in your investments? Secondly, how are you going to mitigate those risks? Then land restoration is one of the key elements.”
I4: “Another part is still fundamentally investing in real assets and land and commodities, which has traditionally been seen as a hedge against inflation and volatility in sort of a low-risk investment strategy for pension funds, where they can get a pretty steady yield, and then potentially uplift from the value of the land on exit. So that's the foundation.” (…) “Because if you're interested in performance, that's one thing, but also if you're interested in just risk and return, you're now realizing that you still want to measure and understand the natural capital to why you're potentially exposed in terms of your returns. I mean, if you'd like a simple example: If you buy an agricultural asset that has no water security in Australia, that's a big risk against your productivity. So I think people are understanding that it needs to be assessed in any case.” (…) “And then the other thing is, I think, for some investors and banks, where they're highly exposed to extracted markets, oil and gas or non+sustainable markets, they want to hedge that risk. But again, from a PR perspective, but also from a pure value perspective. That if those markets get severely regulated it all goes bad, then that hedge will offset if they're invested in this sustainability sector, as well. That's the primal piece that I'm really trying, which I'm not sure anyone's really buying yet but I think it will emerge, I think there's significant uplift in terms of return.”
I7: ”There is a risk element. The central bank, for example, defined that ESG. And not incorporating ESG into your investments is a risk. So if you only focus on the yield, you're getting with a certain investment with a return for certain investments, and not looking at the risk. You're probably doing a bad job. And that goes again, back to the fiduciary duty, basically. So already, like I said, five years ago, whenever people started analyzing their investments also in terms of, okay, what could be the impact of hurricanes or floodings for my investment? Or if I invest into an oil company like Shell, which a lot of the Dutch investors did, what are my risks that I'm investing into stranded assets?”

## Slide 13
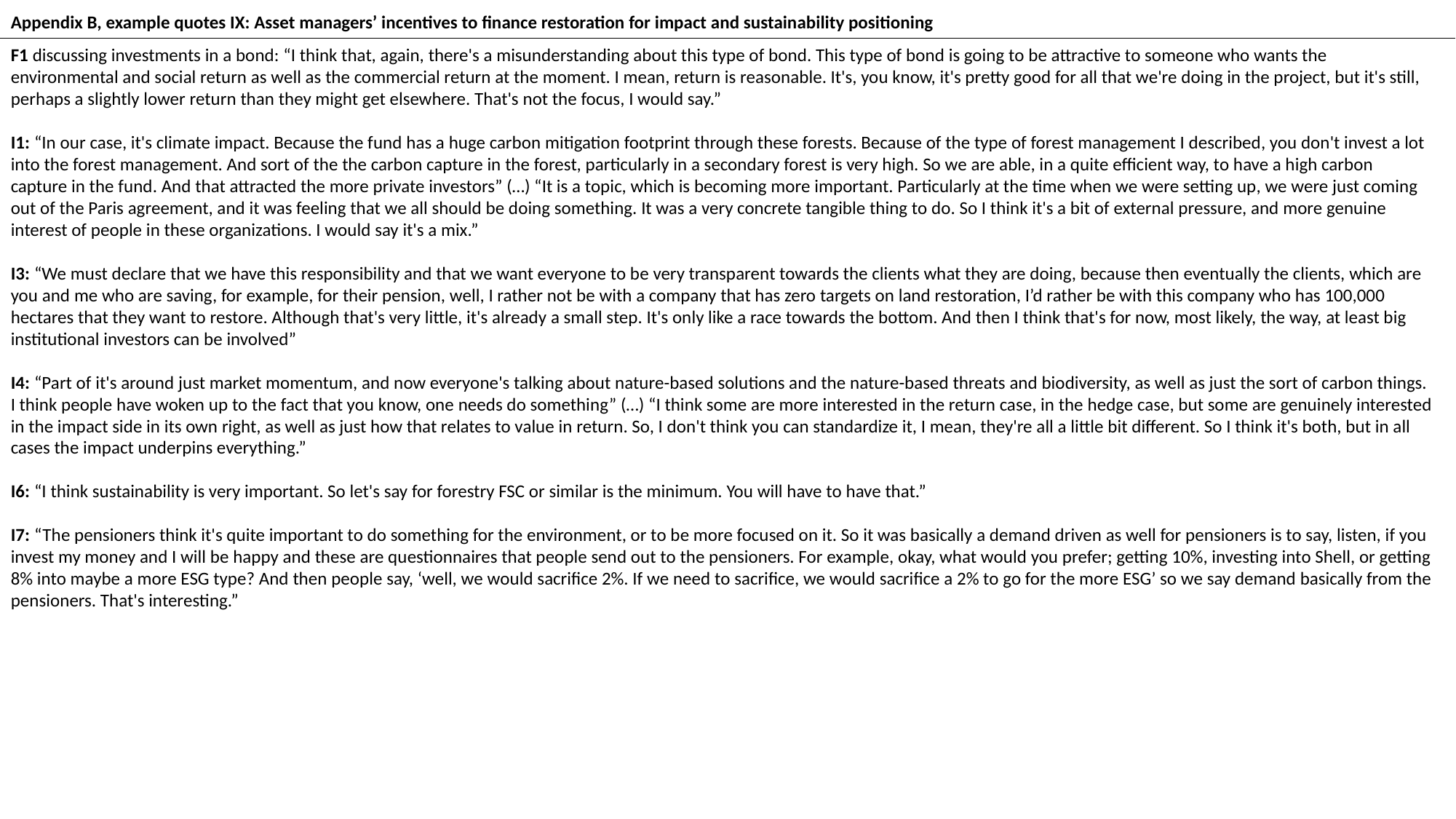

Appendix B, example quotes IX: Asset managers’ incentives to finance restoration for impact and sustainability positioning
F1 discussing investments in a bond: “I think that, again, there's a misunderstanding about this type of bond. This type of bond is going to be attractive to someone who wants the environmental and social return as well as the commercial return at the moment. I mean, return is reasonable. It's, you know, it's pretty good for all that we're doing in the project, but it's still, perhaps a slightly lower return than they might get elsewhere. That's not the focus, I would say.”
I1: “In our case, it's climate impact. Because the fund has a huge carbon mitigation footprint through these forests. Because of the type of forest management I described, you don't invest a lot into the forest management. And sort of the the carbon capture in the forest, particularly in a secondary forest is very high. So we are able, in a quite efficient way, to have a high carbon capture in the fund. And that attracted the more private investors” (…) “It is a topic, which is becoming more important. Particularly at the time when we were setting up, we were just coming out of the Paris agreement, and it was feeling that we all should be doing something. It was a very concrete tangible thing to do. So I think it's a bit of external pressure, and more genuine interest of people in these organizations. I would say it's a mix.”
I3: “We must declare that we have this responsibility and that we want everyone to be very transparent towards the clients what they are doing, because then eventually the clients, which are you and me who are saving, for example, for their pension, well, I rather not be with a company that has zero targets on land restoration, I’d rather be with this company who has 100,000 hectares that they want to restore. Although that's very little, it's already a small step. It's only like a race towards the bottom. And then I think that's for now, most likely, the way, at least big institutional investors can be involved”
I4: “Part of it's around just market momentum, and now everyone's talking about nature-based solutions and the nature-based threats and biodiversity, as well as just the sort of carbon things. I think people have woken up to the fact that you know, one needs do something” (…) “I think some are more interested in the return case, in the hedge case, but some are genuinely interested in the impact side in its own right, as well as just how that relates to value in return. So, I don't think you can standardize it, I mean, they're all a little bit different. So I think it's both, but in all cases the impact underpins everything.”
I6: “I think sustainability is very important. So let's say for forestry FSC or similar is the minimum. You will have to have that.”
I7: “The pensioners think it's quite important to do something for the environment, or to be more focused on it. So it was basically a demand driven as well for pensioners is to say, listen, if you invest my money and I will be happy and these are questionnaires that people send out to the pensioners. For example, okay, what would you prefer; getting 10%, investing into Shell, or getting 8% into maybe a more ESG type? And then people say, ‘well, we would sacrifice 2%. If we need to sacrifice, we would sacrifice a 2% to go for the more ESG’ so we say demand basically from the pensioners. That's interesting.”

## Slide 14
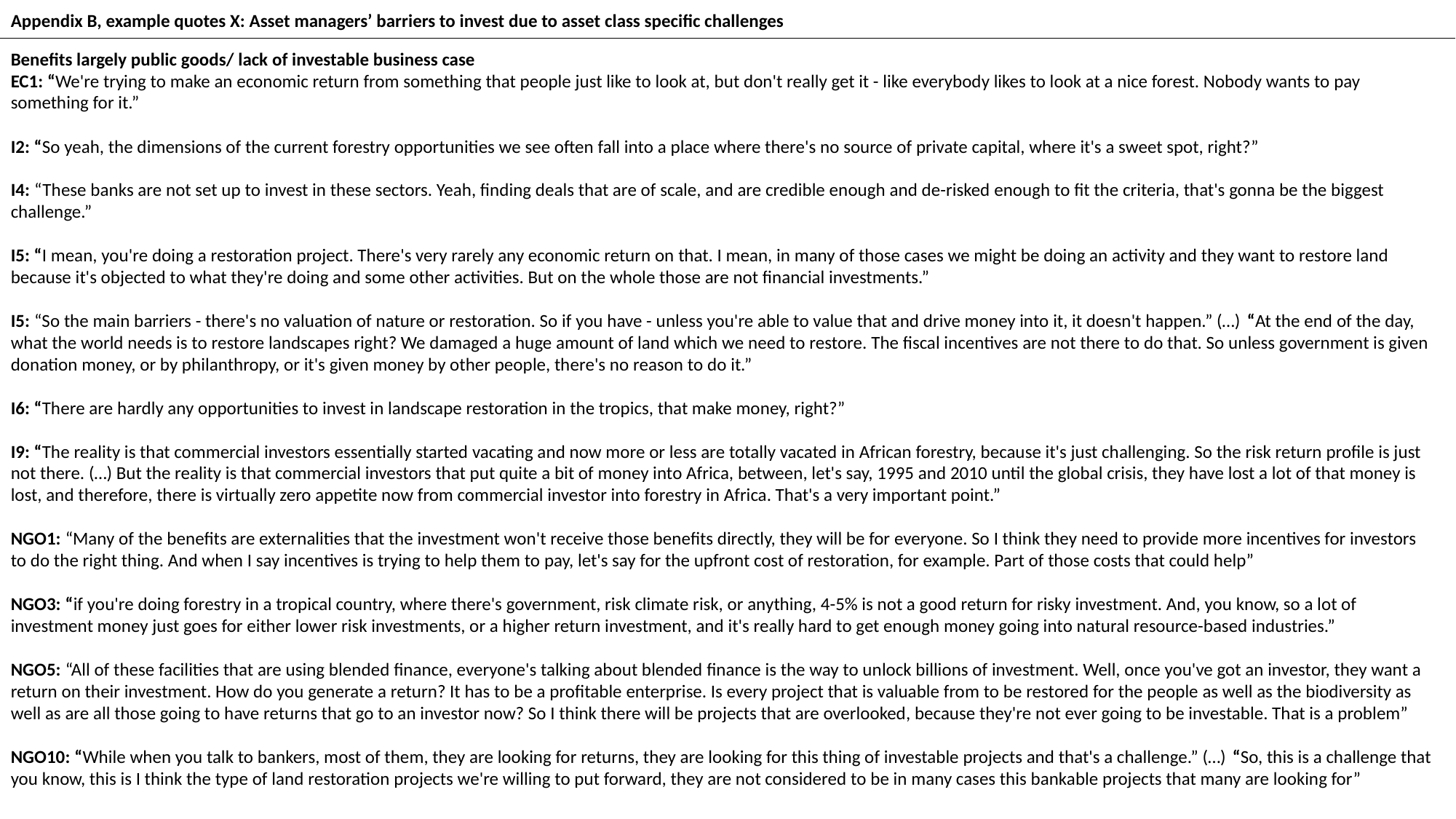

Appendix B, example quotes X: Asset managers’ barriers to invest due to asset class specific challenges
Benefits largely public goods/ lack of investable business case
EC1: “We're trying to make an economic return from something that people just like to look at, but don't really get it - like everybody likes to look at a nice forest. Nobody wants to pay something for it.”
I2: “So yeah, the dimensions of the current forestry opportunities we see often fall into a place where there's no source of private capital, where it's a sweet spot, right?”
I4: “These banks are not set up to invest in these sectors. Yeah, finding deals that are of scale, and are credible enough and de-risked enough to fit the criteria, that's gonna be the biggest challenge.”
I5: “I mean, you're doing a restoration project. There's very rarely any economic return on that. I mean, in many of those cases we might be doing an activity and they want to restore land because it's objected to what they're doing and some other activities. But on the whole those are not financial investments.”
I5: “So the main barriers - there's no valuation of nature or restoration. So if you have - unless you're able to value that and drive money into it, it doesn't happen.” (…) “At the end of the day, what the world needs is to restore landscapes right? We damaged a huge amount of land which we need to restore. The fiscal incentives are not there to do that. So unless government is given donation money, or by philanthropy, or it's given money by other people, there's no reason to do it.”
I6: “There are hardly any opportunities to invest in landscape restoration in the tropics, that make money, right?”
I9: “The reality is that commercial investors essentially started vacating and now more or less are totally vacated in African forestry, because it's just challenging. So the risk return profile is just not there. (…) But the reality is that commercial investors that put quite a bit of money into Africa, between, let's say, 1995 and 2010 until the global crisis, they have lost a lot of that money is lost, and therefore, there is virtually zero appetite now from commercial investor into forestry in Africa. That's a very important point.”
NGO1: “Many of the benefits are externalities that the investment won't receive those benefits directly, they will be for everyone. So I think they need to provide more incentives for investors to do the right thing. And when I say incentives is trying to help them to pay, let's say for the upfront cost of restoration, for example. Part of those costs that could help”
NGO3: “if you're doing forestry in a tropical country, where there's government, risk climate risk, or anything, 4-5% is not a good return for risky investment. And, you know, so a lot of investment money just goes for either lower risk investments, or a higher return investment, and it's really hard to get enough money going into natural resource-based industries.”
NGO5: “All of these facilities that are using blended finance, everyone's talking about blended finance is the way to unlock billions of investment. Well, once you've got an investor, they want a return on their investment. How do you generate a return? It has to be a profitable enterprise. Is every project that is valuable from to be restored for the people as well as the biodiversity as well as are all those going to have returns that go to an investor now? So I think there will be projects that are overlooked, because they're not ever going to be investable. That is a problem”
NGO10: “While when you talk to bankers, most of them, they are looking for returns, they are looking for this thing of investable projects and that's a challenge.” (…) “So, this is a challenge that you know, this is I think the type of land restoration projects we're willing to put forward, they are not considered to be in many cases this bankable projects that many are looking for”

## Slide 15
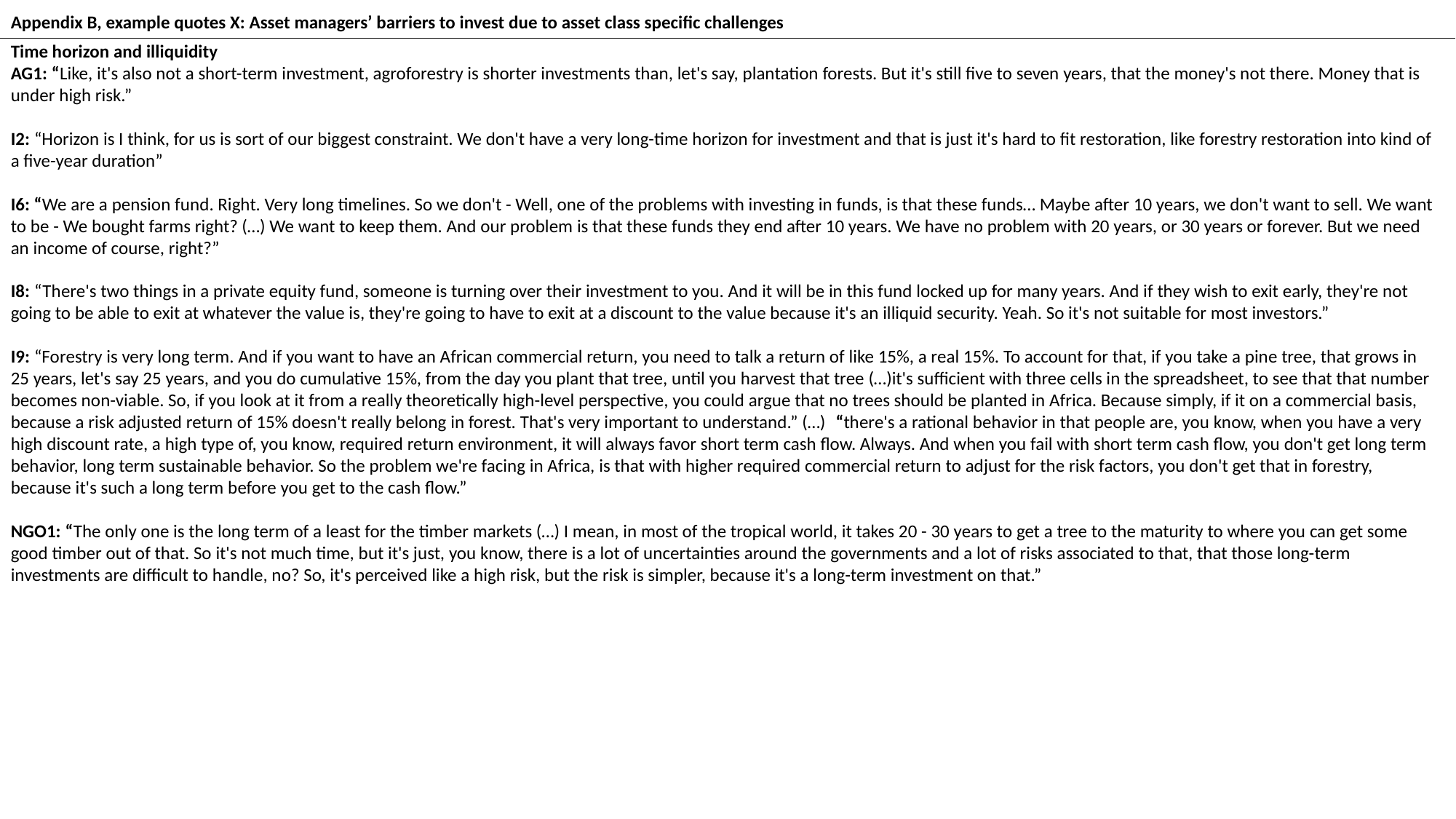

Appendix B, example quotes X: Asset managers’ barriers to invest due to asset class specific challenges
Time horizon and illiquidity
AG1: “Like, it's also not a short-term investment, agroforestry is shorter investments than, let's say, plantation forests. But it's still five to seven years, that the money's not there. Money that is under high risk.”
I2: “Horizon is I think, for us is sort of our biggest constraint. We don't have a very long-time horizon for investment and that is just it's hard to fit restoration, like forestry restoration into kind of a five-year duration”
I6: “We are a pension fund. Right. Very long timelines. So we don't - Well, one of the problems with investing in funds, is that these funds… Maybe after 10 years, we don't want to sell. We want to be - We bought farms right? (…) We want to keep them. And our problem is that these funds they end after 10 years. We have no problem with 20 years, or 30 years or forever. But we need an income of course, right?”
I8: “There's two things in a private equity fund, someone is turning over their investment to you. And it will be in this fund locked up for many years. And if they wish to exit early, they're not going to be able to exit at whatever the value is, they're going to have to exit at a discount to the value because it's an illiquid security. Yeah. So it's not suitable for most investors.”
I9: “Forestry is very long term. And if you want to have an African commercial return, you need to talk a return of like 15%, a real 15%. To account for that, if you take a pine tree, that grows in 25 years, let's say 25 years, and you do cumulative 15%, from the day you plant that tree, until you harvest that tree (…)it's sufficient with three cells in the spreadsheet, to see that that number becomes non-viable. So, if you look at it from a really theoretically high-level perspective, you could argue that no trees should be planted in Africa. Because simply, if it on a commercial basis, because a risk adjusted return of 15% doesn't really belong in forest. That's very important to understand.” (…) “there's a rational behavior in that people are, you know, when you have a very high discount rate, a high type of, you know, required return environment, it will always favor short term cash flow. Always. And when you fail with short term cash flow, you don't get long term behavior, long term sustainable behavior. So the problem we're facing in Africa, is that with higher required commercial return to adjust for the risk factors, you don't get that in forestry, because it's such a long term before you get to the cash flow.”
NGO1: “The only one is the long term of a least for the timber markets (…) I mean, in most of the tropical world, it takes 20 - 30 years to get a tree to the maturity to where you can get some good timber out of that. So it's not much time, but it's just, you know, there is a lot of uncertainties around the governments and a lot of risks associated to that, that those long-term investments are difficult to handle, no? So, it's perceived like a high risk, but the risk is simpler, because it's a long-term investment on that.”

## Slide 16
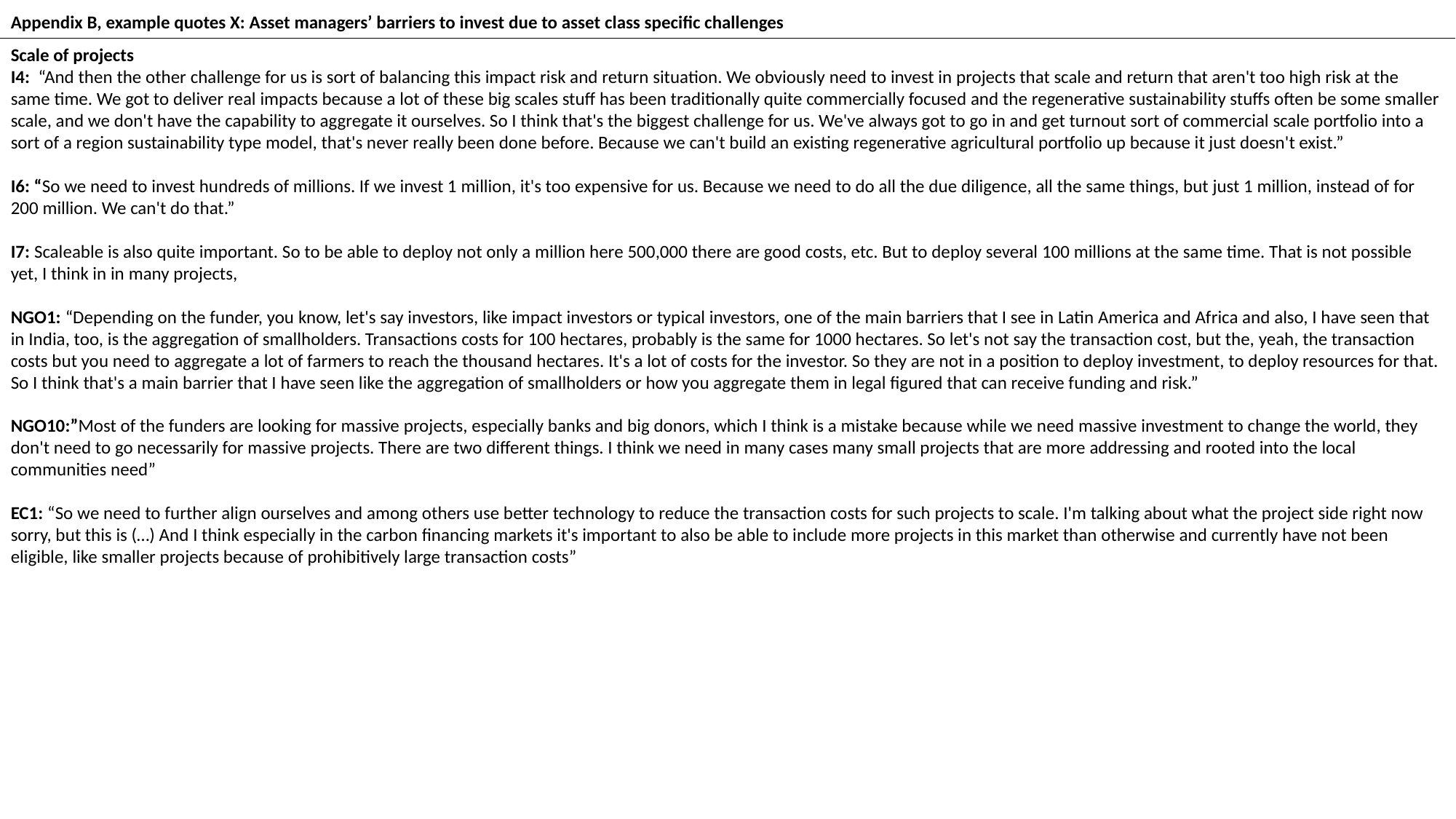

Appendix B, example quotes X: Asset managers’ barriers to invest due to asset class specific challenges
Scale of projects
I4: “And then the other challenge for us is sort of balancing this impact risk and return situation. We obviously need to invest in projects that scale and return that aren't too high risk at the same time. We got to deliver real impacts because a lot of these big scales stuff has been traditionally quite commercially focused and the regenerative sustainability stuffs often be some smaller scale, and we don't have the capability to aggregate it ourselves. So I think that's the biggest challenge for us. We've always got to go in and get turnout sort of commercial scale portfolio into a sort of a region sustainability type model, that's never really been done before. Because we can't build an existing regenerative agricultural portfolio up because it just doesn't exist.”
I6: “So we need to invest hundreds of millions. If we invest 1 million, it's too expensive for us. Because we need to do all the due diligence, all the same things, but just 1 million, instead of for 200 million. We can't do that.”
I7: Scaleable is also quite important. So to be able to deploy not only a million here 500,000 there are good costs, etc. But to deploy several 100 millions at the same time. That is not possible yet, I think in in many projects,
NGO1: “Depending on the funder, you know, let's say investors, like impact investors or typical investors, one of the main barriers that I see in Latin America and Africa and also, I have seen that in India, too, is the aggregation of smallholders. Transactions costs for 100 hectares, probably is the same for 1000 hectares. So let's not say the transaction cost, but the, yeah, the transaction costs but you need to aggregate a lot of farmers to reach the thousand hectares. It's a lot of costs for the investor. So they are not in a position to deploy investment, to deploy resources for that. So I think that's a main barrier that I have seen like the aggregation of smallholders or how you aggregate them in legal figured that can receive funding and risk.”
NGO10:”Most of the funders are looking for massive projects, especially banks and big donors, which I think is a mistake because while we need massive investment to change the world, they don't need to go necessarily for massive projects. There are two different things. I think we need in many cases many small projects that are more addressing and rooted into the local communities need”
EC1: “So we need to further align ourselves and among others use better technology to reduce the transaction costs for such projects to scale. I'm talking about what the project side right now sorry, but this is (…) And I think especially in the carbon financing markets it's important to also be able to include more projects in this market than otherwise and currently have not been eligible, like smaller projects because of prohibitively large transaction costs”

## Slide 17
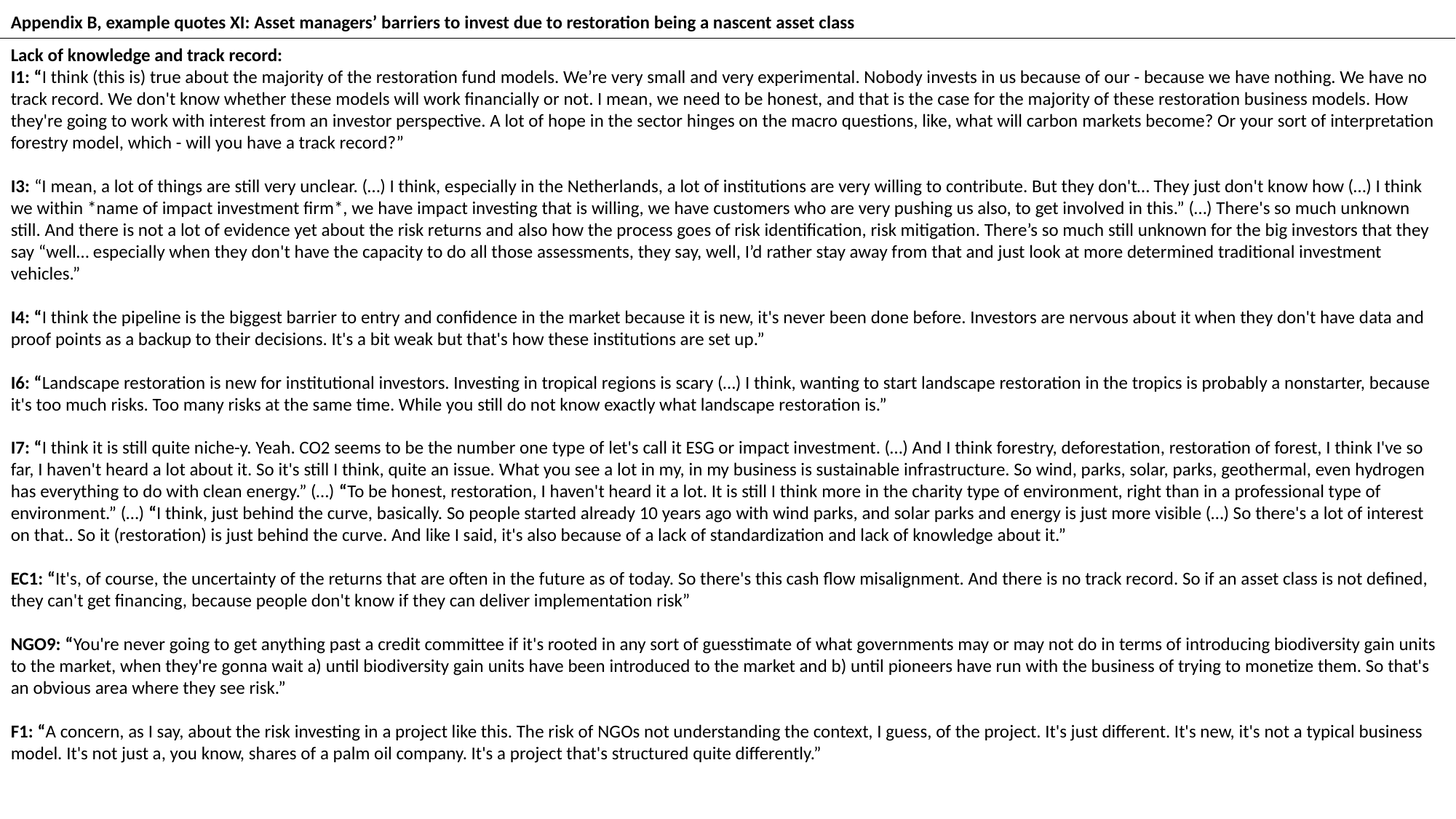

Appendix B, example quotes XI: Asset managers’ barriers to invest due to restoration being a nascent asset class
Lack of knowledge and track record:
I1: “I think (this is) true about the majority of the restoration fund models. We’re very small and very experimental. Nobody invests in us because of our - because we have nothing. We have no track record. We don't know whether these models will work financially or not. I mean, we need to be honest, and that is the case for the majority of these restoration business models. How they're going to work with interest from an investor perspective. A lot of hope in the sector hinges on the macro questions, like, what will carbon markets become? Or your sort of interpretation forestry model, which - will you have a track record?”
I3: “I mean, a lot of things are still very unclear. (…) I think, especially in the Netherlands, a lot of institutions are very willing to contribute. But they don't… They just don't know how (…) I think we within *name of impact investment firm*, we have impact investing that is willing, we have customers who are very pushing us also, to get involved in this.” (…) There's so much unknown still. And there is not a lot of evidence yet about the risk returns and also how the process goes of risk identification, risk mitigation. There’s so much still unknown for the big investors that they say “well… especially when they don't have the capacity to do all those assessments, they say, well, I’d rather stay away from that and just look at more determined traditional investment vehicles.”
I4: “I think the pipeline is the biggest barrier to entry and confidence in the market because it is new, it's never been done before. Investors are nervous about it when they don't have data and proof points as a backup to their decisions. It's a bit weak but that's how these institutions are set up.”
I6: “Landscape restoration is new for institutional investors. Investing in tropical regions is scary (…) I think, wanting to start landscape restoration in the tropics is probably a nonstarter, because it's too much risks. Too many risks at the same time. While you still do not know exactly what landscape restoration is.”
I7: “I think it is still quite niche-y. Yeah. CO2 seems to be the number one type of let's call it ESG or impact investment. (…) And I think forestry, deforestation, restoration of forest, I think I've so far, I haven't heard a lot about it. So it's still I think, quite an issue. What you see a lot in my, in my business is sustainable infrastructure. So wind, parks, solar, parks, geothermal, even hydrogen has everything to do with clean energy.” (…) “To be honest, restoration, I haven't heard it a lot. It is still I think more in the charity type of environment, right than in a professional type of environment.” (…) “I think, just behind the curve, basically. So people started already 10 years ago with wind parks, and solar parks and energy is just more visible (…) So there's a lot of interest on that.. So it (restoration) is just behind the curve. And like I said, it's also because of a lack of standardization and lack of knowledge about it.”
EC1: “It's, of course, the uncertainty of the returns that are often in the future as of today. So there's this cash flow misalignment. And there is no track record. So if an asset class is not defined, they can't get financing, because people don't know if they can deliver implementation risk”
NGO9: “You're never going to get anything past a credit committee if it's rooted in any sort of guesstimate of what governments may or may not do in terms of introducing biodiversity gain units to the market, when they're gonna wait a) until biodiversity gain units have been introduced to the market and b) until pioneers have run with the business of trying to monetize them. So that's an obvious area where they see risk.”
F1: “A concern, as I say, about the risk investing in a project like this. The risk of NGOs not understanding the context, I guess, of the project. It's just different. It's new, it's not a typical business model. It's not just a, you know, shares of a palm oil company. It's a project that's structured quite differently.”

## Slide 18
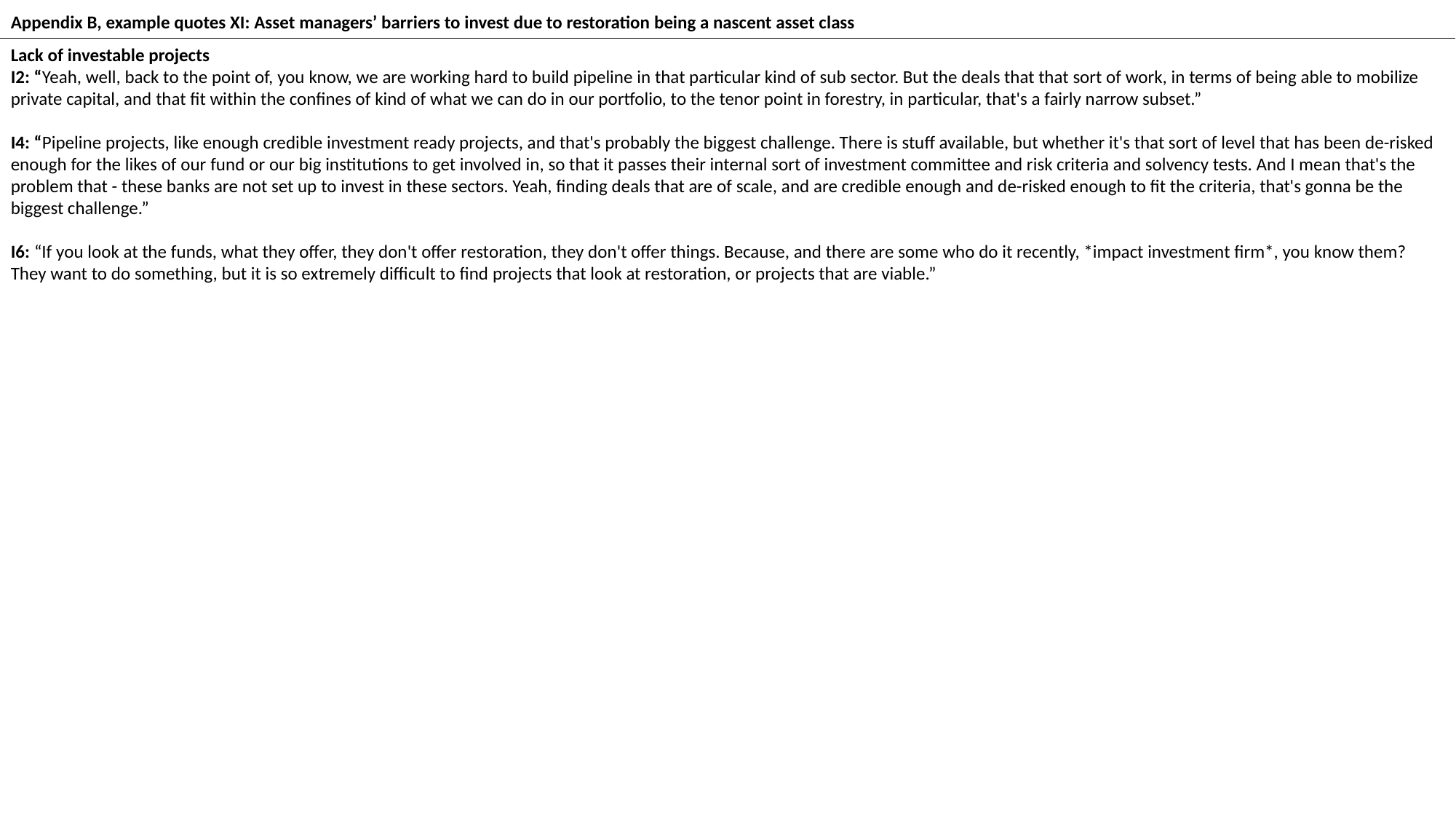

Appendix B, example quotes XI: Asset managers’ barriers to invest due to restoration being a nascent asset class
Lack of investable projects
I2: “Yeah, well, back to the point of, you know, we are working hard to build pipeline in that particular kind of sub sector. But the deals that that sort of work, in terms of being able to mobilize private capital, and that fit within the confines of kind of what we can do in our portfolio, to the tenor point in forestry, in particular, that's a fairly narrow subset.”
I4: “Pipeline projects, like enough credible investment ready projects, and that's probably the biggest challenge. There is stuff available, but whether it's that sort of level that has been de-risked enough for the likes of our fund or our big institutions to get involved in, so that it passes their internal sort of investment committee and risk criteria and solvency tests. And I mean that's the problem that - these banks are not set up to invest in these sectors. Yeah, finding deals that are of scale, and are credible enough and de-risked enough to fit the criteria, that's gonna be the biggest challenge.”
I6: “If you look at the funds, what they offer, they don't offer restoration, they don't offer things. Because, and there are some who do it recently, *impact investment firm*, you know them? They want to do something, but it is so extremely difficult to find projects that look at restoration, or projects that are viable.”

## Slide 19
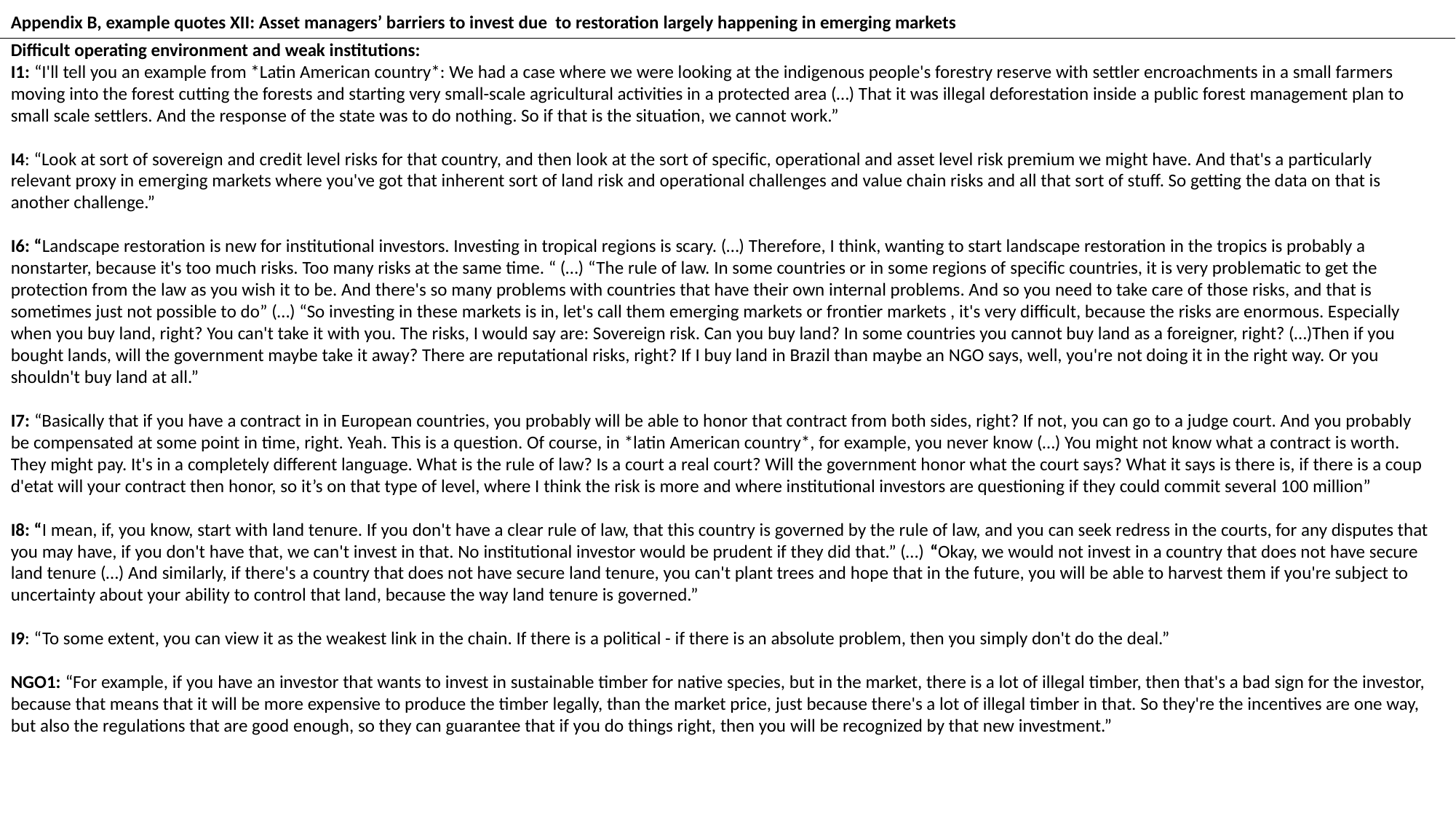

Appendix B, example quotes XII: Asset managers’ barriers to invest due to restoration largely happening in emerging markets
Difficult operating environment and weak institutions:
I1: “I'll tell you an example from *Latin American country*: We had a case where we were looking at the indigenous people's forestry reserve with settler encroachments in a small farmers moving into the forest cutting the forests and starting very small-scale agricultural activities in a protected area (…) That it was illegal deforestation inside a public forest management plan to small scale settlers. And the response of the state was to do nothing. So if that is the situation, we cannot work.”
I4: “Look at sort of sovereign and credit level risks for that country, and then look at the sort of specific, operational and asset level risk premium we might have. And that's a particularly relevant proxy in emerging markets where you've got that inherent sort of land risk and operational challenges and value chain risks and all that sort of stuff. So getting the data on that is another challenge.”
I6: “Landscape restoration is new for institutional investors. Investing in tropical regions is scary. (…) Therefore, I think, wanting to start landscape restoration in the tropics is probably a nonstarter, because it's too much risks. Too many risks at the same time. “ (…) “The rule of law. In some countries or in some regions of specific countries, it is very problematic to get the protection from the law as you wish it to be. And there's so many problems with countries that have their own internal problems. And so you need to take care of those risks, and that is sometimes just not possible to do” (…) “So investing in these markets is in, let's call them emerging markets or frontier markets , it's very difficult, because the risks are enormous. Especially when you buy land, right? You can't take it with you. The risks, I would say are: Sovereign risk. Can you buy land? In some countries you cannot buy land as a foreigner, right? (…)Then if you bought lands, will the government maybe take it away? There are reputational risks, right? If I buy land in Brazil than maybe an NGO says, well, you're not doing it in the right way. Or you shouldn't buy land at all.”
I7: “Basically that if you have a contract in in European countries, you probably will be able to honor that contract from both sides, right? If not, you can go to a judge court. And you probably be compensated at some point in time, right. Yeah. This is a question. Of course, in *latin American country*, for example, you never know (…) You might not know what a contract is worth. They might pay. It's in a completely different language. What is the rule of law? Is a court a real court? Will the government honor what the court says? What it says is there is, if there is a coup d'etat will your contract then honor, so it’s on that type of level, where I think the risk is more and where institutional investors are questioning if they could commit several 100 million”
I8: “I mean, if, you know, start with land tenure. If you don't have a clear rule of law, that this country is governed by the rule of law, and you can seek redress in the courts, for any disputes that you may have, if you don't have that, we can't invest in that. No institutional investor would be prudent if they did that.” (…) “Okay, we would not invest in a country that does not have secure land tenure (…) And similarly, if there's a country that does not have secure land tenure, you can't plant trees and hope that in the future, you will be able to harvest them if you're subject to uncertainty about your ability to control that land, because the way land tenure is governed.”
I9: “To some extent, you can view it as the weakest link in the chain. If there is a political - if there is an absolute problem, then you simply don't do the deal.”
NGO1: “For example, if you have an investor that wants to invest in sustainable timber for native species, but in the market, there is a lot of illegal timber, then that's a bad sign for the investor, because that means that it will be more expensive to produce the timber legally, than the market price, just because there's a lot of illegal timber in that. So they're the incentives are one way, but also the regulations that are good enough, so they can guarantee that if you do things right, then you will be recognized by that new investment.”

## Slide 20
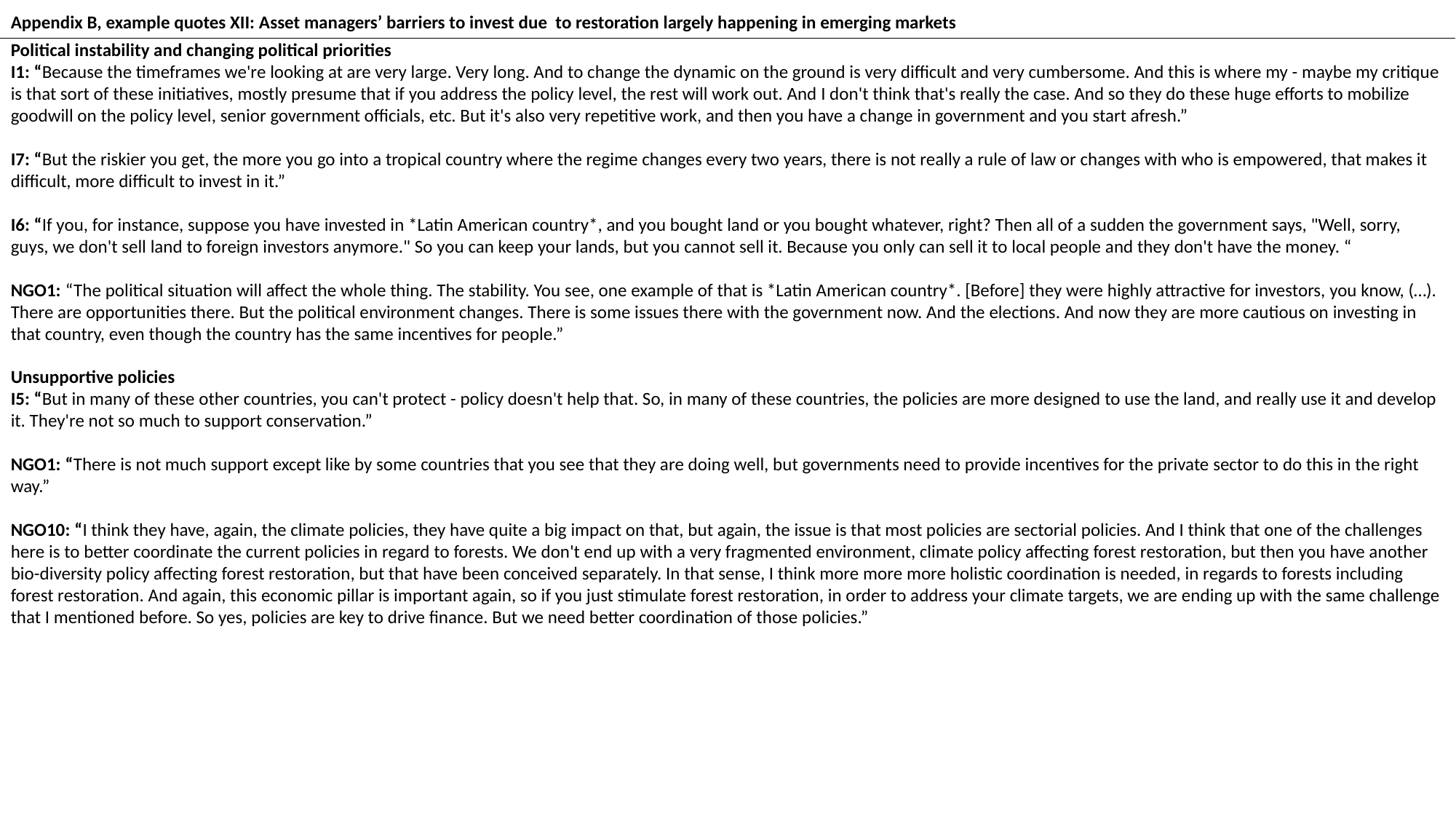

Appendix B, example quotes XII: Asset managers’ barriers to invest due to restoration largely happening in emerging markets
Political instability and changing political priorities
I1: “Because the timeframes we're looking at are very large. Very long. And to change the dynamic on the ground is very difficult and very cumbersome. And this is where my - maybe my critique is that sort of these initiatives, mostly presume that if you address the policy level, the rest will work out. And I don't think that's really the case. And so they do these huge efforts to mobilize goodwill on the policy level, senior government officials, etc. But it's also very repetitive work, and then you have a change in government and you start afresh.”
I7: “But the riskier you get, the more you go into a tropical country where the regime changes every two years, there is not really a rule of law or changes with who is empowered, that makes it difficult, more difficult to invest in it.”
I6: “If you, for instance, suppose you have invested in *Latin American country*, and you bought land or you bought whatever, right? Then all of a sudden the government says, "Well, sorry, guys, we don't sell land to foreign investors anymore." So you can keep your lands, but you cannot sell it. Because you only can sell it to local people and they don't have the money. “
NGO1: “The political situation will affect the whole thing. The stability. You see, one example of that is *Latin American country*. [Before] they were highly attractive for investors, you know, (…). There are opportunities there. But the political environment changes. There is some issues there with the government now. And the elections. And now they are more cautious on investing in that country, even though the country has the same incentives for people.”
Unsupportive policies
I5: “But in many of these other countries, you can't protect - policy doesn't help that. So, in many of these countries, the policies are more designed to use the land, and really use it and develop it. They're not so much to support conservation.”
NGO1: “There is not much support except like by some countries that you see that they are doing well, but governments need to provide incentives for the private sector to do this in the right way.”
NGO10: “I think they have, again, the climate policies, they have quite a big impact on that, but again, the issue is that most policies are sectorial policies. And I think that one of the challenges here is to better coordinate the current policies in regard to forests. We don't end up with a very fragmented environment, climate policy affecting forest restoration, but then you have another bio-diversity policy affecting forest restoration, but that have been conceived separately. In that sense, I think more more more holistic coordination is needed, in regards to forests including forest restoration. And again, this economic pillar is important again, so if you just stimulate forest restoration, in order to address your climate targets, we are ending up with the same challenge that I mentioned before. So yes, policies are key to drive finance. But we need better coordination of those policies.”

## Slide 21
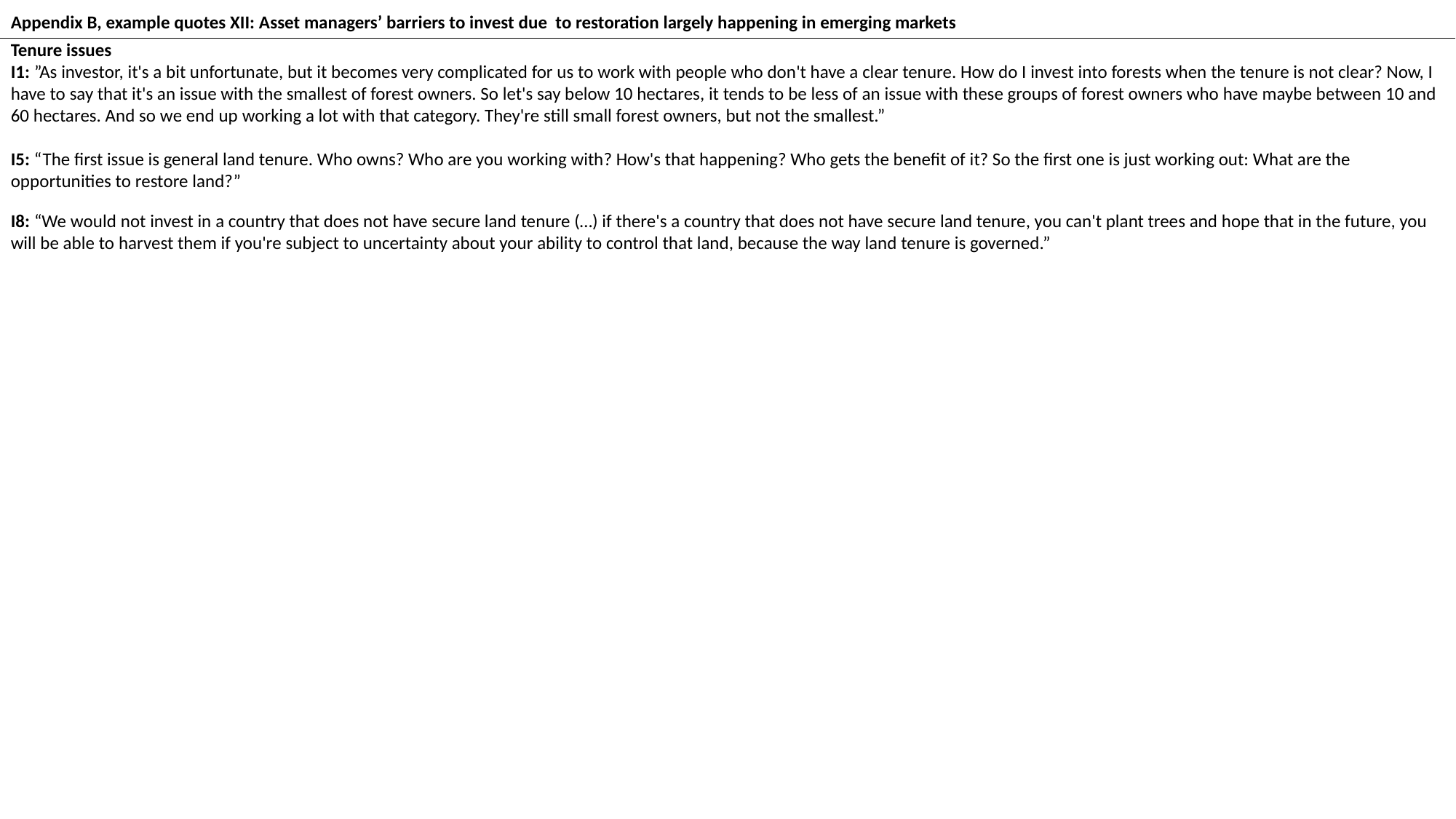

Appendix B, example quotes XII: Asset managers’ barriers to invest due to restoration largely happening in emerging markets
Tenure issues
I1: ”As investor, it's a bit unfortunate, but it becomes very complicated for us to work with people who don't have a clear tenure. How do I invest into forests when the tenure is not clear? Now, I have to say that it's an issue with the smallest of forest owners. So let's say below 10 hectares, it tends to be less of an issue with these groups of forest owners who have maybe between 10 and 60 hectares. And so we end up working a lot with that category. They're still small forest owners, but not the smallest.”
I5: “The first issue is general land tenure. Who owns? Who are you working with? How's that happening? Who gets the benefit of it? So the first one is just working out: What are the opportunities to restore land?”
I8: “We would not invest in a country that does not have secure land tenure (…) if there's a country that does not have secure land tenure, you can't plant trees and hope that in the future, you will be able to harvest them if you're subject to uncertainty about your ability to control that land, because the way land tenure is governed.”

## Slide 22
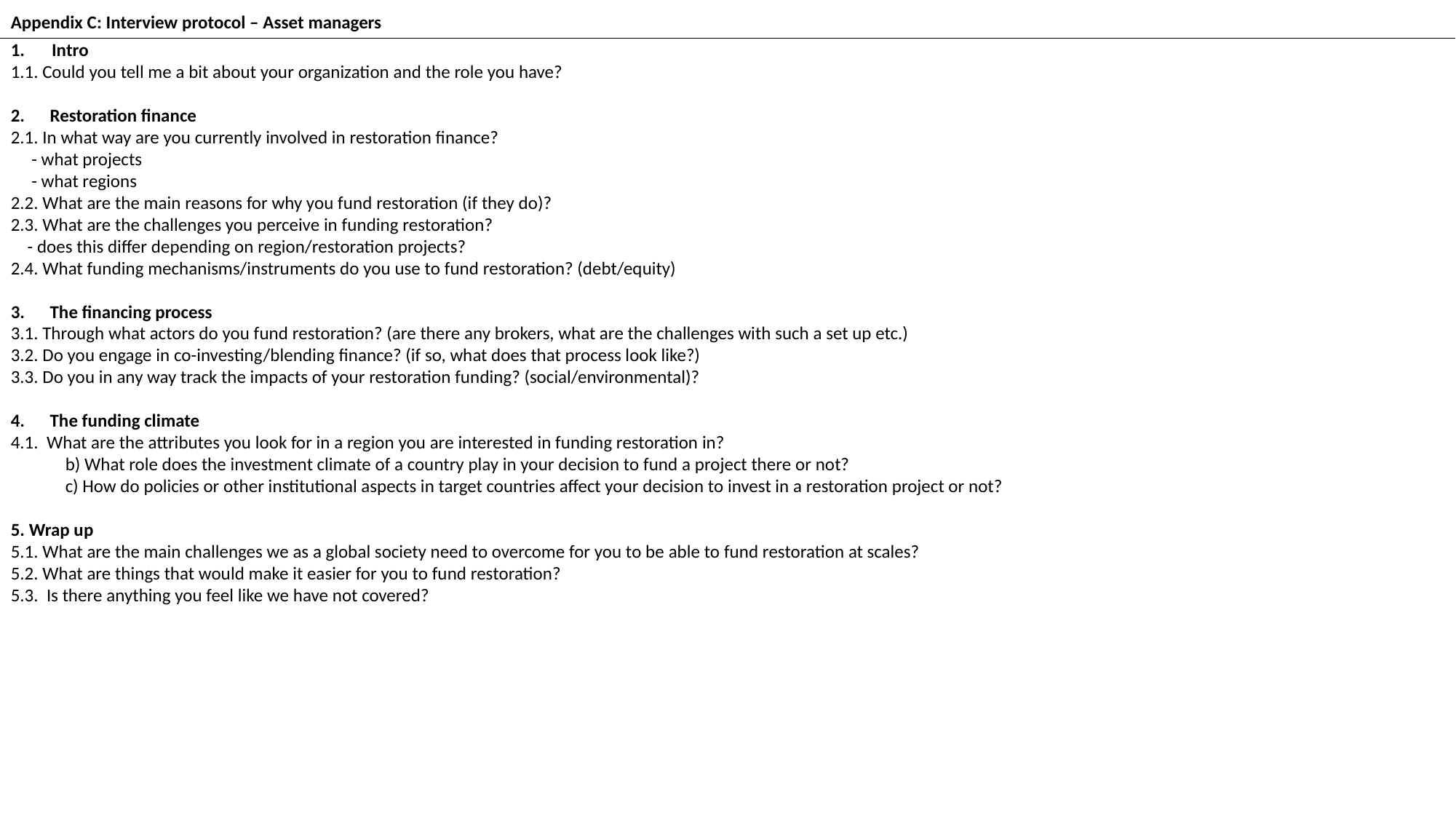

Appendix C: Interview protocol – Asset managers
Intro
1.1. Could you tell me a bit about your organization and the role you have?
2. Restoration finance
2.1. In what way are you currently involved in restoration finance?
 - what projects
 - what regions
2.2. What are the main reasons for why you fund restoration (if they do)?
2.3. What are the challenges you perceive in funding restoration?
 - does this differ depending on region/restoration projects?
2.4. What funding mechanisms/instruments do you use to fund restoration? (debt/equity)
3. The financing process
3.1. Through what actors do you fund restoration? (are there any brokers, what are the challenges with such a set up etc.)
3.2. Do you engage in co-investing/blending finance? (if so, what does that process look like?)
3.3. Do you in any way track the impacts of your restoration funding? (social/environmental)?
4. The funding climate
4.1. What are the attributes you look for in a region you are interested in funding restoration in?
b) What role does the investment climate of a country play in your decision to fund a project there or not?
c) How do policies or other institutional aspects in target countries affect your decision to invest in a restoration project or not?
5. Wrap up
5.1. What are the main challenges we as a global society need to overcome for you to be able to fund restoration at scales?
5.2. What are things that would make it easier for you to fund restoration?
5.3. Is there anything you feel like we have not covered?

## Slide 23
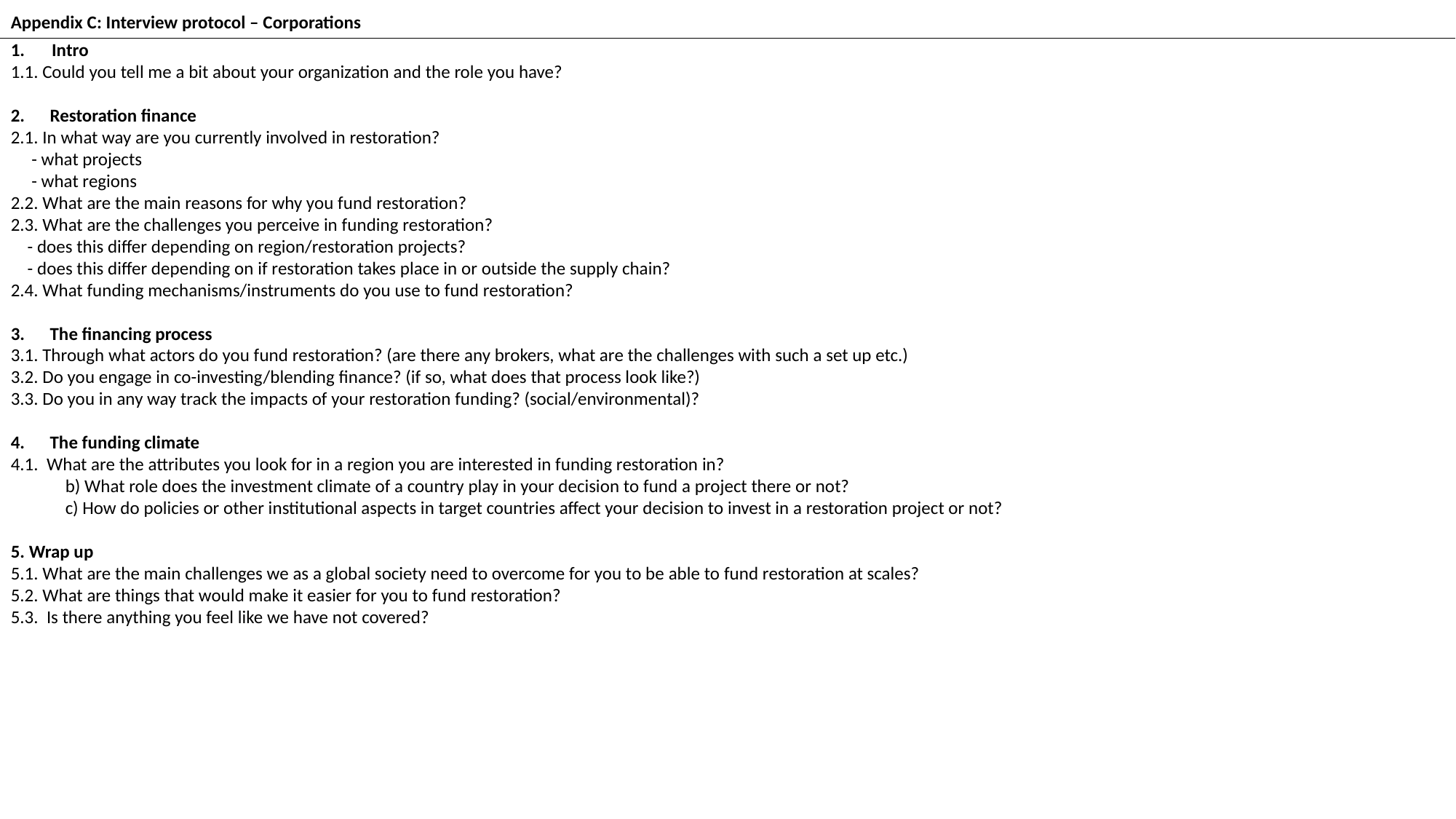

Appendix C: Interview protocol – Corporations
Intro
1.1. Could you tell me a bit about your organization and the role you have?
2. Restoration finance
2.1. In what way are you currently involved in restoration?
 - what projects
 - what regions
2.2. What are the main reasons for why you fund restoration?
2.3. What are the challenges you perceive in funding restoration?
 - does this differ depending on region/restoration projects?
 - does this differ depending on if restoration takes place in or outside the supply chain?
2.4. What funding mechanisms/instruments do you use to fund restoration?
3. The financing process
3.1. Through what actors do you fund restoration? (are there any brokers, what are the challenges with such a set up etc.)
3.2. Do you engage in co-investing/blending finance? (if so, what does that process look like?)
3.3. Do you in any way track the impacts of your restoration funding? (social/environmental)?
4. The funding climate
4.1. What are the attributes you look for in a region you are interested in funding restoration in?
b) What role does the investment climate of a country play in your decision to fund a project there or not?
c) How do policies or other institutional aspects in target countries affect your decision to invest in a restoration project or not?
5. Wrap up
5.1. What are the main challenges we as a global society need to overcome for you to be able to fund restoration at scales?
5.2. What are things that would make it easier for you to fund restoration?
5.3. Is there anything you feel like we have not covered?

## Slide 24
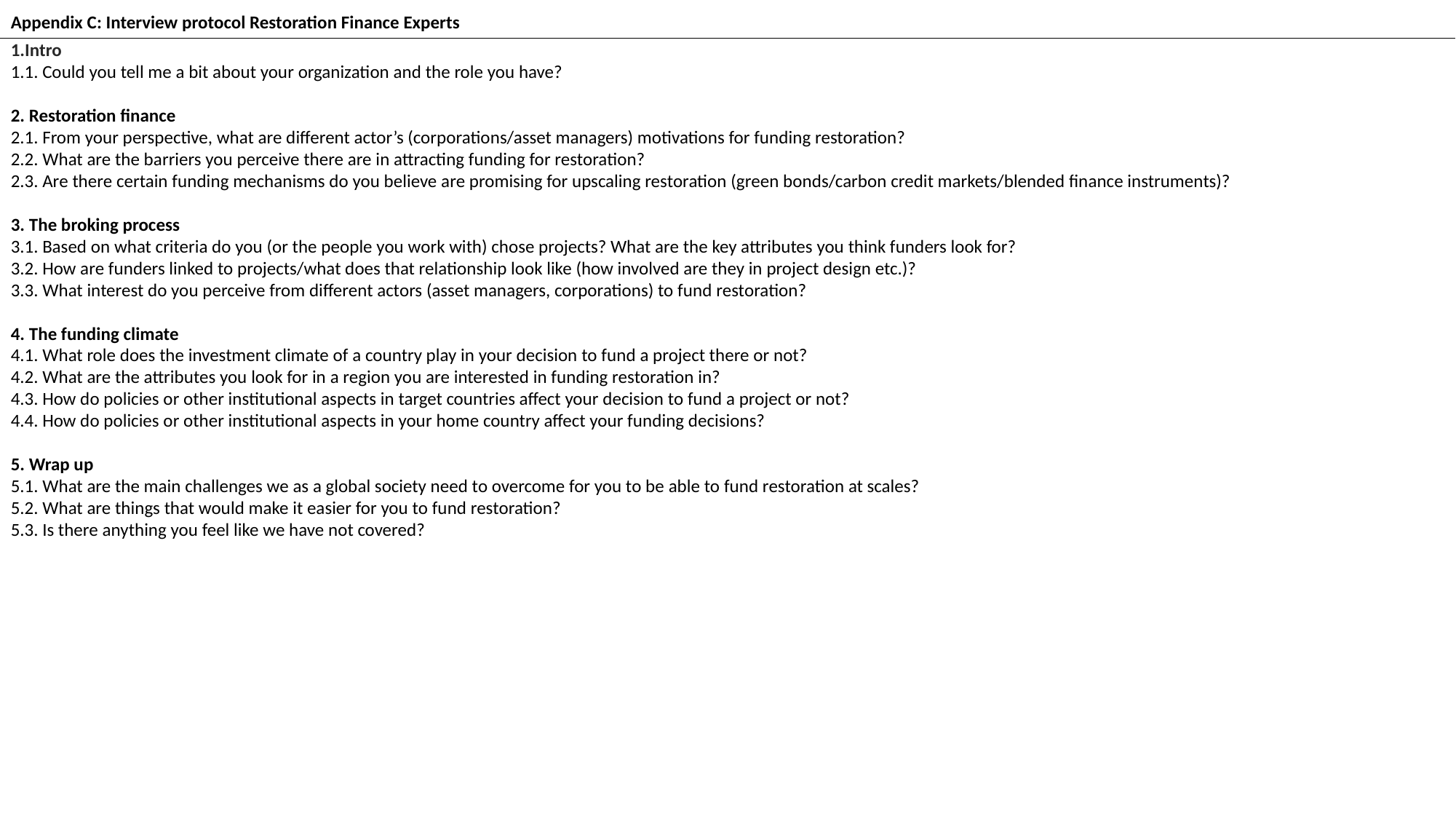

Appendix C: Interview protocol Restoration Finance Experts
1.Intro
1.1. Could you tell me a bit about your organization and the role you have?
2. Restoration finance
2.1. From your perspective, what are different actor’s (corporations/asset managers) motivations for funding restoration?
2.2. What are the barriers you perceive there are in attracting funding for restoration?
2.3. Are there certain funding mechanisms do you believe are promising for upscaling restoration (green bonds/carbon credit markets/blended finance instruments)?
3. The broking process
3.1. Based on what criteria do you (or the people you work with) chose projects? What are the key attributes you think funders look for?
3.2. How are funders linked to projects/what does that relationship look like (how involved are they in project design etc.)?
3.3. What interest do you perceive from different actors (asset managers, corporations) to fund restoration?
4. The funding climate
4.1. What role does the investment climate of a country play in your decision to fund a project there or not?
4.2. What are the attributes you look for in a region you are interested in funding restoration in?
4.3. How do policies or other institutional aspects in target countries affect your decision to fund a project or not?
4.4. How do policies or other institutional aspects in your home country affect your funding decisions?
5. Wrap up
5.1. What are the main challenges we as a global society need to overcome for you to be able to fund restoration at scales?
5.2. What are things that would make it easier for you to fund restoration?
5.3. Is there anything you feel like we have not covered?
