# Supplementary material for: Incentives and barriers to private finance for forest and landscape restoration
Source: Nat Ecol Evol. 2023 May 8;7(5):707–15. doi: 10.1038/s41559-023-02037-5 (PMC10172125; doi:10.1038/s41559-023-02037-5)
Supplement: Supplementary file 2 — Reporting Summary [file 41559_2023_2037_MOESM2_ESM.pdf]

nature portfolio

Corresponding author(s): Sara Löfqvist, Rachael D. Garrett, Jaboury Ghazoul

Last updated by author(s): Jan 8, 2023

Reporting Summary

Nature Portfolio wishes to improve the reproducibility of the work that we publish. This form provides structure for consistency and transparency in reporting. For further information on Nature Portfolio policies, see our Editorial Policies and the Editorial Policy Checklist.

Statistics

For all statistical analyses, confirm that the following items are present in the figure legend, table legend, main text, or Methods section.

n/a	Confirmed	
☒	☐	The exact sample size (*n*) for each experimental group/condition, given as a discrete number and unit of measurement
☒	☐	A statement on whether measurements were taken from distinct samples or whether the same sample was measured repeatedly
☒	☐	The statistical test(s) used AND whether they are one- or two-sided<br>*Only common tests should be described solely by name; describe more complex techniques in the Methods section.*
☒	☐	A description of all covariates tested
☒	☐	A description of any assumptions or corrections, such as tests of normality and adjustment for multiple comparisons
☒	☐	A full description of the statistical parameters including central tendency (e.g. means) or other basic estimates (e.g. regression coefficient) AND variation (e.g. standard deviation) or associated estimates of uncertainty (e.g. confidence intervals)
☒	☐	For null hypothesis testing, the test statistic (e.g. F, t, r) with confidence intervals, effect sizes, degrees of freedom and P value noted<br>*Give P values as exact values whenever suitable.*
☒	☐	For Bayesian analysis, information on the choice of priors and Markov chain Monte Carlo settings
☒	☐	For hierarchical and complex designs, identification of the appropriate level for tests and full reporting of outcomes
☒	☐	Estimates of effect sizes (e.g. Cohen's *d*, Pearson's *r*), indicating how they were calculated

Our web collection on statistics for biologists contains articles on many of the points above.

Software and code

Policy information about availability of computer code

Data collection	No software was used
Data analysis	The transcription software Otter was used for transcribing interviews, and NVivo was used for data analysis.

For manuscripts utilizing custom algorithms or software that are central to the research but not yet described in published literature, software must be made available to editors and reviewers. We strongly encourage code deposition in a community repository (e.g. GitHub). See the Nature Portfolio guidelines for submitting code & software for further information.

Data

Policy information about availability of data

All manuscripts must include a data availability statement. This statement should provide the following information, where applicable:

- Accession codes, unique identifiers, or web links for publicly available datasets
- A description of any restrictions on data availability
- For clinical datasets or third party data, please ensure that the statement adheres to our policy

Codes representing each respondent is available in the manuscript. In the supplementary material each statement presentated in the paper is listed together with a list of codes who made that statement. Examples of quotes backing up each statement is also available in the supplementary material. All data is made available through Figshare.

Human research participants

Policy information about studies involving human research participants and Sex and Gender in Research.

Reporting on sex and gender	We did not consider gender identity or sex in this study, as we were solely looking for the participants perspectives from the point of view of their professional roles. In the assessment of restoration finance potential we believe that gender identity and sex is not a key factor influencing behavior or perspectives, so we did not ask respondents to report it.
Population characteristics	See answer above. As we focused on professional perspectives we did not account for population characteristic beyond those potentially affecting professional perspectives, which we identified to be area in which participant's organization is located, what type of organization they work for, and which role they have in that organization.
Recruitment	The interviewees were sampled using existing networks, snowball sampling, and from attendee list from relevant events (such as GLF Luxembourgs' finance session 2019). We acknowledge the self-selection bias that our population is subject to, and that asset managers and corporations with interest in restoration may have been more likely to be interested in participating in the study. The self-selection bias in our study may increase the risk that we got a too positive outlook on potential from private finance to fund restoration, as our interviewees a)were likely interested in the topic of restoration, and b) had an interest in presenting their organization as pro-sustainable solutions. See more on how we addressed this bias under "research sample" on the next page.
Ethics oversight	The ethics comission at ETH Zurich approved the study

Note that full information on the approval of the study protocol must also be provided in the manuscript.

Field-specific reporting

Please select the one below that is the best fit for your research. If you are not sure, read the appropriate sections before making your selection.

☐ Life sciences ☒ Behavioural & social sciences ☐ Ecological, evolutionary & environmental sciences

For a reference copy of the document with all sections, see nature.com/documents/nr-reporting-summary-flat.pdf

Behavioural & social sciences study design

All studies must disclose on these points even when the disclosure is negative.

Study description	The study is qualitative and data was collected through semi-structured interviews. The data was analyzed in the analyzing software NVivo through thematic analysis in which key themes across respondents were identified inductively. We mixed thematic analysis with inductive methods, meaning that we started out with a conceptual framing of relevant components and interaction, but then made smaller adjustments to the interview guide as the interview proceeded and new themes emerged. The aim with this method was to gain a rich and nuanced understanding of the issue we study, given the lack of prior research that has been conducted on this topic.
Research sample	The research sample consists of 9 representatives from asset management firms, 7 representatives from corporations, 10 representatives from NGOs, 2 environmental consultants, 1 representative from a conservation focused foundation, and 1 agroforestry initiative. These actors hold diverse expertise in the topic of restoration finance, and the diversity of interviewees gave us a well-rounded understanding of the topic of restoration finance. We acknowledge the self-selection bias that our population is subject to, and that asset managers and corporations with interest in restoration may have been more likely to be interested in participating in the study. We mitigate this bias by including interviewees who work with private funders (especially the NGOs and the environmental consultants), to indirectly capture perspectives of a wider array of private funding actors, and also to capture a more critical view on private actors engagement in restoration. Another bias is linked to the possible incentive of participants to present themselves as more sustainability oriented than they actually are. We tended to this bias by providing anonymity to interviewees, and further on in the interview process, by bringing up potentially more sensitive topics ourselves. In this way, we would mention that a certain perspective had come up in prior interviews and ask if participants had any experience of this themselves. In this way we aimed to lower the barriers to discussing topics that had been identified as relevant but that could be percieved as sensitive. Yet, we acknowledge that the actors that have no interest in restoration finance, and are not collaborating with NGOs or consultants on other sustainability matters were not captured by our study.
Sampling strategy	The interviewees were sampled using existing networks, snowball sampling, and from attendee list from relevant events (such as GLF Luxembourgs' finance session 2019). We did not determine sample size at the onset of the project, but instead continued sampling interviewees and conducting interviews until saturation across key themes was reached. That is, we ended data collection when new interviews did not provide insights that had not been captured by prior interviews.
Data collection	Data was collected through semi-structured interviews. The interviews were conducted over zoom, Skype, or Microsoft teams, and recorded with the zoom recording and/or with the iPhone voice recorder. Notes were taken throughout the interviews. In all but one interview, the researcher conducted the interview alone, and in one interview a supervising researcher participated. The researcher conducting the interviews had been involved in designing the study and was aware of the research design and questions when

	conducting the interviews. Prior to the interview the participants received a one pager explaining the project, together with an information sheet from the IRB.
Timing	The data was collected between March 2020 and December 2021
Data exclusions	Two interviews were excluded from the study. Both those interviews were with impact investors. One was excluded because the outcome of the interview did not fit the research questions, instead another person from the same firm was later interviewed. The second interview was excluded because the transcription was lost.
Non-participation	No participants dropped out after agreeing to the study. Approximately 25% of respondents that were contacted agreed to be interviewed, the others did not respond to interview requests.
Randomization	Participants were not allocated into experimental groups.

Reporting for specific materials, systems and methods

We require information from authors about some types of materials, experimental systems and methods used in many studies. Here, indicate whether each material, system or method listed is relevant to your study. If you are not sure if a list item applies to your research, read the appropriate section before selecting a response.

Materials & experimental systems

n/a	Involved in the study
☒	☐ Antibodies
☒	☐ Eukaryotic cell lines
☒	☐ Palaeontology and archaeology
☒	☐ Animals and other organisms
☒	☐ Clinical data
☒	☐ Dual use research of concern

Methods

n/a	Involved in the study
☒	☐ ChIP-seq
☒	☐ Flow cytometry
☒	☐ MRI-based neuroimaging
